# Cosolvent electrolyte chemistries for high-voltage potassium-ion battery

**DOI:** 10.1093/nsr/nwae359

**Published:** 2024-10-15

**Authors:** Mengkang Shen, Zhongqin Dai, Ling Fan, Hongwei Fu, Yuanhui Geng, Jie Guan, Fanfei Sun, Apparao M Rao, Jiang Zhou, Bingan Lu

**Affiliations:** School of Physics and Electronics, Hunan University, Changsha 410082, China; School of Physical Science and Technology, ShanghaiTech University, Shanghai 201210, China; School of Physics and Electronics, Hunan University, Changsha 410082, China; School of Physics and Electronics, Hunan University, Changsha 410082, China; School of Physics and Electronics, Hunan University, Changsha 410082, China; School of Physics and Electronics, Hunan University, Changsha 410082, China; Shanghai Synchrotron Radiation Facility, Shanghai Advanced Research Institute, Chinese Academy of Sciences, Shanghai 201204, China; Shanghai Institute of Applied Physics, Chinese Academy of Sciences, Shanghai 201204, China; Department of Physics and Astronomy, Clemson Nanomaterials Institute, Clemson University, Clemson SC 29634, USA; School of Materials Science and Engineering, Central South University, Changsha 410083, China; School of Physics and Electronics, Hunan University, Changsha 410082, China; State Key Laboratory of Advanced Design and Manufacturing for Vehicle Body, Hunan University, Changsha 410082, China

**Keywords:** potassium-ion battery, cosolvent electrolyte, high-voltage, anion-rich solvation, large solvation clusters

## Abstract

The poor oxidation resistance of traditional electrolytes has hampered the development of high-voltage potassium-ion battery technology. Here, we present a cosolvent electrolyte design strategy to overcome the high-voltage limitations of potassium-ion electrolyte chemistries. The cosolvent electrolyte breaks the dissolution limitation of the salt through ion–dipole interactions, significantly enlarging the anion-rich solvation clusters, as verified by the *insitu* synchrotron-based wide-angle X-ray scattering experiments. Furthermore, the large anion-rich solvation clusters also facilitate the formation of an effective electrode–electrolyte interphase, thereby enhancing compatibility with high-voltage electrodes. The cosolvent electrolyte enables K||Prussian blue cells (2–4.5 V) to operate for >700 cycles with a capacity retention of 91.9%. Our cosolvent electrolyte design strategy paves new avenues for the development of high-voltage potassium-ion batteries and beyond.

## Teaser

This study utilized the cosolvency phenomenon to design an electrolyte that significantly enlarges the anion-rich solvation clusters, thereby facilitating the formation of a beneficial electrode-electrolyte interface and improving high-voltage compatibility.

## INTRODUCTION

Potassium-ion batteries (PIBs) have shown excellent prospects for large-scale energy storage due to their cost-effectiveness, resource abundance and potential high-voltage window [1–3]. The electrolyte type is particularly critical for battery performance due to its dominant role in forming the all-important electrode–electrolyte interphase [4,5]. Generally, ether-based electrolytes have attracted much attention due to their good potassium salt solubility and high ionic conductivity [6,7]. However, ether-based electrolytes have shortcomings, such as poor oxidation resistance and incompatibility with graphite anodes, which limits their application in graphite-based or high-voltage batteries [8,9].

High-concentration electrolytes (HCE) can significantly reduce the number of free solvent molecules in the electrolyte, thereby promoting the formation of more contact ion pairs (CIPs) and aggregated clusters (AGGs) [10,11]. This strategy effectively broadens the electrochemical window and promotes the formation of an anions-dominated solid–electrolyte interphase (SEI), significantly improving battery performance. However, the increased electrolyte viscosity and costly HCE have hampered its large-scale practical application [12,13]. The localized high-concentration electrolyte (LHCE) was developed by introducing diluent to HCE, with the salt concentration and viscosity decreased. At the same time, the CIPs and AGGs solvation structures and their characteristics in HCE are expected to be retained [14]. However, the LHCE suffers from salt precipitation issues when a large amount of diluent is introduced (especially in potassium-ion electrolytes, as shown in Fig. S1), decreasing the salt concentration and reducing the AGGs proportion, which adversely affect the electrochemical window [15,16]. Moreover, the use of fluorine-containing diluents in conventional LHCE systems is expensive as well as a potential environmental hazard, which runs counter to the current emphasis on ecologically benign and sustainable development in battery technology [17,18]. In considering these factors, future LHCE research must explore non-fluorinated diluents that do not significantly reduce salt solubility and AGGs while promoting their high-voltage properties.

The cosolvency property in certain mixed solvent systems can greatly improve the solubility of the solutes when the proportion of each component is adjusted precisely [19,20]. It is based on the solvation structure transformation that is induced by the synergy between solvent molecules, leading to a more efficient dissolution of the target compound [21,22]. Cosolvency is particularly important in the pharmaceuticals [23] and several combinations of cosolvents include water and alcohols (such as ethanol, propylene glycol), mixtures of polyethylene glycol and other organic solvents, etc. [24,25]. For batteries, cosolvency can enhance the dissolution of salts, thereby promoting more anions to enter the primary solvation shell and increase AGGs. This has a direct bearing on optimizing and reconstructing the microstructure of the electrolyte, which is essential for designing high-voltage electrolytes [26,27]. It is of great significance to explore the underlying mechanisms of cosolvency and design electrolyte formulations based on these for battery systems.

Here, we propose cosolvent electrolyte by introducing 1,2-dibutoxyethane (DBE) into supersaturated potassium bis(fluorosulfonyl)imide (KFSI)–diglyme (DGM) electrolytes, which significantly enlarges the anion-rich solvation clusters and is conducive to the formation of an effective electrode–electrolyte interphase and improves high-voltage compatibility. By utilizing this cosolvent electrolyte, the K||Prussian blue (PB [28]) cells (2–4.5 V) operate stably for >700 cycles with an average coulombic efficiency of ≤99.2%. This work cleverly utilizes the cosolvency phenomenon to innovate a strategy for electrolyte engineering to promote technological progress in the field of high-voltage PIBs.

## RESULTS AND DISCUSSION

### Cosolvent electrolyte design strategy

**Figure 1.**
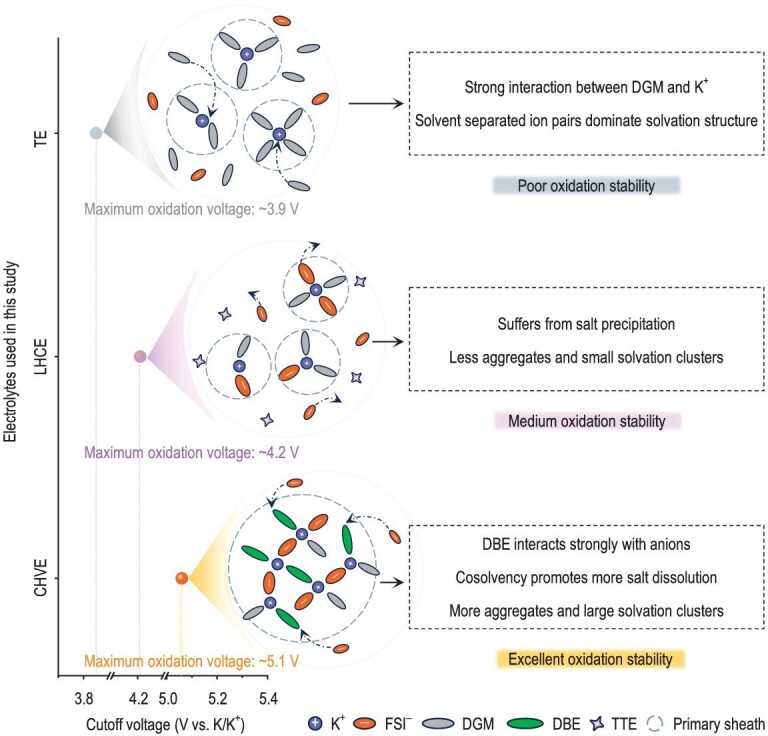
Design concept of cosolvent electrolyte.

Three types of comparative electrolyte systems were constructed, including a traditional electrolyte (TE, 1.5 M KFSI in DGM), an LHCE (1.5 M KFSI in DGM–TTE) with 1,1,2,2-tetrafluoroethyl-2,2,3,3-tetrafluoropropylether (TTE) as the diluent and a cosolvent high-voltage electrolyte (CHVE, 1.5 M KFSI in DGM–DBE) with DBE as the cosolvent. The specific electrolyte configuration information is presented in Table S1. Although CHVE has a low ion conductivity (probably because of the formation of larger clusters), it is still sufficient to meet the normal operation conditions for the battery (Fig. S2). In TE, the strong interaction between DGM and K^+^ will lead to a prevalent solvent-separated ion pairs (SSIPs) solvation structure, restricting its oxidation stability (Fig. 1). In LHCE, the introduction of TTE into the high-concentration electrolyte can reduce viscosity and increase ion transport [29,30]. However, the high proportion of TTE will result in severe salt precipitation (especially in potassium-based electrolytes) because TTE does not participate in the solvation, which reduces the salt concentration and AGGs. Additionally, the enhancement of oxidative stability is also restricted (Fig. 1). In contrast, CHVE not only successfully increased the solubility limit of salt beyond the average solubility of two mixed solvents, but also showed a strong affinity between the cosolvent DBE and anions. Apart from directly participating in the creation of the first solvated shell, DBE promoted the involvement of more anions (will be discussed latter), leading to more AGGs and large anion-rich solvation clusters. Finally, the unique solvation structure of CHVE resulted in excellent oxidation stability that was significantly superior to those of the TE and LHCE systems (Fig. 1).

### Verification of the cosolvency phenomenon

**Figure 2.**
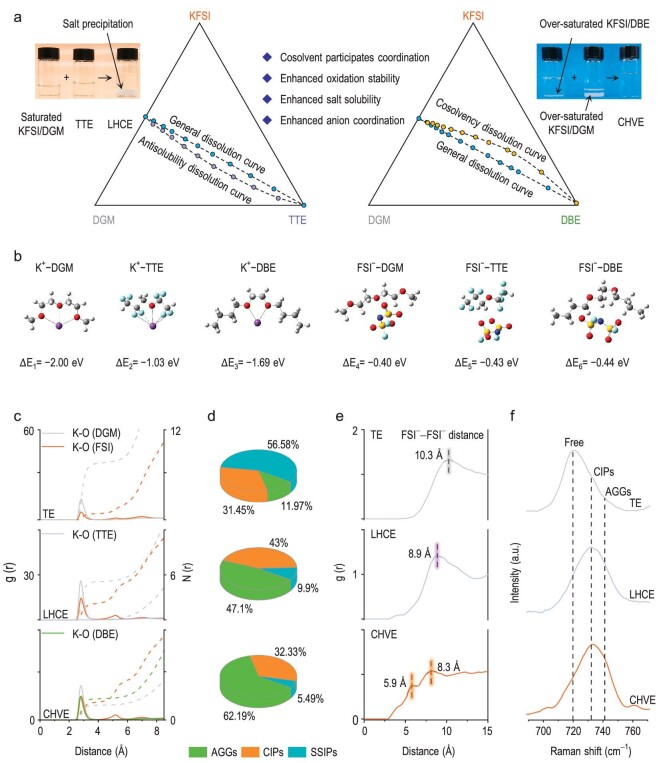
Verification of cosolvency phenomenon and calculation and experimental study of solvation structure. (a) Ternary phase diagram connecting the three variable phases: KFSI, DGM and diluent/cosolvent (TTE/DBE). (b) The binding energies between ions (K^+^ or FSI^−^) and solvent molecules (DGM, TTE or DBE). (c) Radial distribution function calculated by MD simulation of TE, LHCE and CHVE. (d) Distributions of possible primary solvation sheath compositions of the three electrolytes. (e) The RDF of FSI^−^–FSI^−^ in the three electrolytes, derived from MD simulations. (f) Raman spectra of different electrolytes in the range of 690–770 cm^−1^.

The influence of solvent compositional changes on the dissolution characteristics of KFSI salt was investigated by constructing ternary phase diagrams consisting of the KFSI salt, DGM solvent and TTE diluent or DBE cosolvent (Fig. 2a). The KFSI solubility in a single solvent was investigated and it was found that its solubility in DGM (∼6.5 M) is higher than in the TTE diluent (near 0 M) and the DBE cosolvent (∼0.05 M) (Fig. S3). Intuitively, the solubility of a solute in a mixed solvent is the weighted average of its solubility in each solvent (blue dotted lines in Fig. 2a). However, when the TTE diluent is present in the mixed solvent, it significantly decreases the solubility of the KFSI (purple dotted line in Fig. 2a and Table S2), as verified by the precipitated KFSI in the optical image on the left in Fig. 2a. In contrast, when the cosolvent DBE is present in the mixed solvent system, the solubility of the KFSI is enhanced (orange dotted-line data in Fig. 2a and Table S3) and a clear and transparent electrolyte (optical photograph on the right in Fig. 2a) is obtained even if some KFSI salt is present initially in the supersaturated DGM and DBE solvents. Thus, mixing of the two promotes (i) the solubility of the KFSI as discussed above, (ii) AGG formation and (iii) larger solvation clusters, as discussed below. Collectively, cosolvency has a favorable effect on battery performance [27].

Next, the binding energies between ions (K^+^ or FSI^−^) and solvent molecules (DGM, TTE or DBE) were calculated to elucidate the cosolvency mechanism (Fig. 2b). Evidently, the interaction between K^+^ and DGM is stronger (Δ*E* = −2.00 eV) than K^+^–DBE (Δ*E* = −1.69 eV) and K^+^–TTE (Δ*E* = −1.03 eV). Clearly, the interaction between K^+^ and TTE is very weak, which may be one of the reasons why KFSI salts have limited solubility in TTE solvents. Also, the interaction between FSI^−^ and DBE is slightly stronger (Δ*E* = −0.44 eV) than FSI^−^–DGM (Δ*E* = −0.40 eV) and FSI^−^–TTE (Δ*E* = −0.43 eV) and similar results are obtained when the anion (or cation) is completely surrounded by solvent molecules (Fig. S4). These results indicate that DBE is a weakly solvating solvent for cations and a strongly solvating solvent for anions. Thus, in the CHVE system that contains both DGM and DBE, the DGM promotes association with K^+^ while DBE stabilizes and maintains the dissolution state of FSI^−^, leading to an increased solubility of KFSI.

The K^+^ solvation environments in the three electrolytes (TE, LHCE and CHVE) were further analysed through molecular dynamics (MD) simulations (using the LAMMPS package [31], Gaussian16 C0.1 [32] and Multiwfn package [33]) (Figs S5 and S6) and the corresponding radial distribution functions (RDF) of K^+^ are presented in Fig. 2c. Clearly, the average coordination number N(*r*) of K–O_DGM_ is in the order of TE > LHCE > CHVE, while that for the K–O_anion_ is in the opposite trend of TE < LHCE < CHVE (Table S4), which is indicative of the participation of more anions in the solvation sheath in the CHVE system that is beneficial to battery performance [34–36]. Noticeably, the coordination number of K–O_TTE_ in LHCE is close to zero while K–O_DBE_ in CHVE is much higher than that in K–O_DGM_ (Table S4), indicating that the TTE diluent does not participate in the primary solvation sheath whereas the DBE cosolvent does. Furthermore, the proportions of SSIPs, CIPs and AGGs in the three electrolytes were evaluated (Fig. 2d). As expected, the solvation structure in the TE is dominated by SSIPs (56.58%) and CIPs (31.45%), which is consistent with the binding energy and RDF results. Additionally, the proportions of CIPs (43%) and AGGs (47.1%) in LHCE are higher than those in TE, indicating the beneficial role of the TTE diluent in forming an anion-rich solvation structure. By contrast, the AGGs (62.19%) and CIPs (32.33%) are prevalent in the CHVE system, demonstrating that more anions are involved in the solvation structure and they tend to form large anion-rich solvation clusters, which further contributes to battery performance [37].

Furthermore, the RDF of FSI^−^–FSI^−^ reveals a primary peak situated at 10.3 Å in TE (Fig. 2e), indicating the good dispersibility of FSI^−^ anions in the system. However, the RDF of FSI^−^–FSI^−^ exhibits a noteworthy peak at 8.9 Å in LHCE, suggesting a slight aggregation of FSI^−^ anions. By contrast, two main peaks appear at 5.9 and 8.3 Å in CHVE, suggesting the existence of densely anions aggregates and the formation of large compact clusters. The solvation structure of the three electrolytes was also evaluated by using Raman spectroscopy (Fig. 2f). Specifically, the Raman peak of ∼720 cm^–^^1^ is indicative of free anions, while those of ∼730 and ∼740 cm^–^^1^ correspond to CIPs and AGGs, respectively [38]. Clearly, more free anions exist in TE (more SSIPs), while CIPs and AGGs are predominant in LHCE and CHVE, consistently with the computation results discussed above.

### Mesoscopic structure of the cosolvent electrolyte

**Figure 3.**
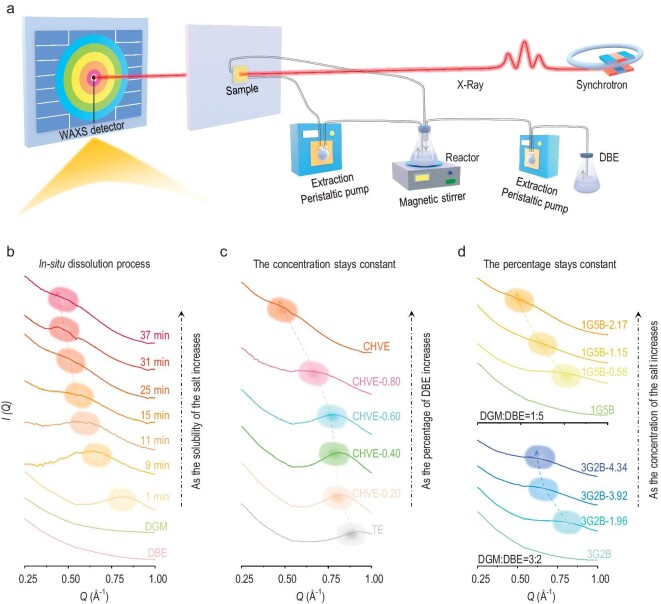
Meso- and microscopic solvation structures. (a) Schematic of the experimental set-up for the *insitu* WAXS experiments. (b) Evolution of the WAXS curves of salt dissolution from 1 to 37 min. (c) The changes in WAXS curves when controlling the amount of salt to remain unchanged and changing the DBE content. The trialing numbers in each legend that labels each curve represent the volume percentages of DBE. (d) The variation of WAXS curves with increasing KFSI concentration under different DGM:DBE ratios. The trialing numbers in each legend that labels each curve represent the moles of soluble KFSI.

To further analyse the microscopic structure of CHVE, we employed a synchrotron-based wide-angle X-ray scattering (WAXS) technique (Fig. S7). Firstly, in order to monitor the solvation structure evolution in real time during the KFSI dissolution in CHVE, we constructed a simple experimental device that included a magnetic stirrer and peristaltic pumps to ensure the precise control of experimental conditions and continuous data collection. A schematic diagram of our *insitu* experimental set-up is shown in Fig. 3a. By using a peristaltic pump, DBE is pumped into the saturated DGM solution with excess KFSI and then the nanoscale structural evolution of CHVE is monitored in real time as the dissolution of KFSI progresses. With gradual dissolution, the peak that is located at 0.81 Å^−1^ in the spectrum shifts gradually towards lower *Q* values (Fig. 3b), indicating an increase in cluster size (diameter: *D* = 2*π*/*Q*) [39–41], consistently with the results discussed above. Moreover, at a constant KFSI concentration (1.5 M), the increasing amount of DBE in CHVE leads to an increasing cluster size in the electrolyte (Fig. 3c). When the ratio of DGM to DBE remains unchanged, the increasing salt concentration also promotes an increasing cluster size (Fig. 3d and Fig. S8). The KFSI concentrations in the mixed DGM and DBE electrolytes are displayed in Table S5. Briefly, CHVE reveals a solvation structure with large anion-rich clusters, which is conducive to obtaining better battery performance, such as an effective electrode–electrolyte interphase and enhanced high-voltage stability. Therefore, the rational utilization of the cosolvent to promote the formation of large solvation clusters is beneficial for the design of high-performance electrolytes.

### Cathode performance and characterization

**Figure 4.**
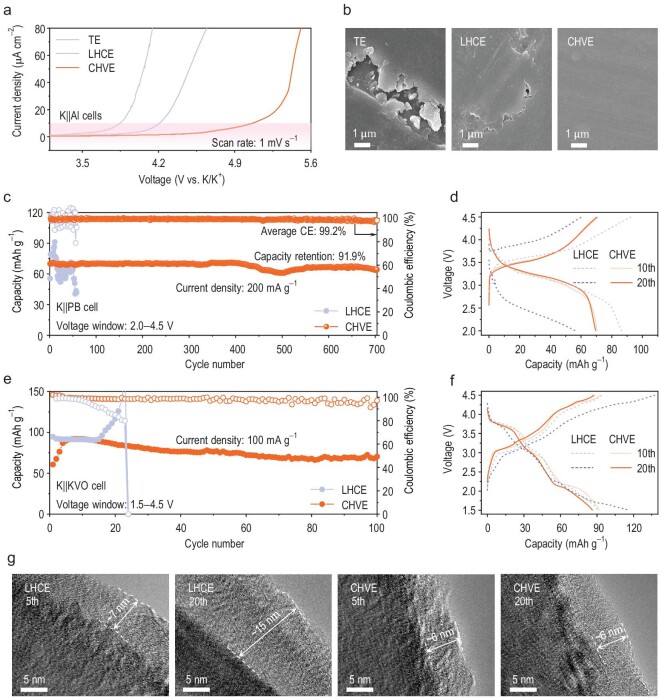
Electrochemical performance and interfacial stability of CHVE. (a) Linear sweep voltammetry profiles of the three electrolytes. (b) Corrosion of aluminum foil under different electrolyte conditions. (c) K||PB cycle performance and (d) charge–discharge curves of the cells with CHVE and LHCE. (e) K||KVO cycle performance and (f) charge–discharge curves of the cells with CHVE and LHCE. (g) TEM images of KVO electrode with different cycle numbers for CHVE and LHCE.

Linear sweep voltammetry (LSV) of K||Al cells under various electrolyte systems was conducted to investigate their stability against aluminum collectors under high-voltage conditions (Fig. 4a). Obviously, CHVE exhibits excellent oxidation stability with a threshold of ≤5.1 V, significantly surpassing TE (3.9 V) and LHCE (4.2 V). Also, aluminum corrosion experiments were performed by using the K||Al cells at a constant voltage of 4.5 V. Compared with the scanning electron microscopy (SEM) images of pristine Al foil (Fig. S9), severe corrosion with large surface cracks on the Al foil were observed with TE (Fig. 4b and Fig. S10), indicating a severe Al corrosion phenomenon. Mild Al corrosion is observed (Fig. 4b and Fig. S11) with LHCE, implying that the TTE diluent is conducive to suppressing the Al corrosion. By contrast, Al foil with CHVE exhibits negligible corrosion, as confirmed by the smooth and flat morphology shown in Fig. 4b and Fig. S12. Thus, the CHVE can effectively suppress the Al corrosion under high-voltage conditions, ensuring the possibility of developing stable high-voltage potassium batteries.

Due to the inferior oxidation voltage of TE, the subsequent discussion that pertains to cathode matching and performance testing will mainly focus on LHCE and CHVE. In the range of 2–4.5 V, the K||PB cells in CHVE exhibited an excellent capacity retention of 91.9% after 700 cycles with an average coulombic efficiency of 99.2% (Fig. 4c). However, the K||PB cells in LHCE suffered a large fluctuation in specific capacity and coulombic efficiency before failing after 50 cycles, probably due to the insufficient oxidation stability under the high cut-off voltage of 4.5 V. The charge–discharge profiles in Fig. 4d attest to the superior high-voltage performance of K||PB in CHVE compared with that in LHCE, which is attributed to the large solvation cluster structure in CHVE (Fig. S13). In addition, in the range of 1.5–4.5 V, the K||δ-K_0.51_V_2_O_5_ (KVO [42]) cells with CHVE deliver 100 stable cycles with good coulombic efficiency (Fig. 4e) that is superior than that with LHCE (∼20 cycles and inferior coulombic efficiency). Compared with the overcharged phenomenon exhibited by K||KVO with LHCE, a good overlap of different cycles was observed with CHVE (Fig. 4f), once again verifying its excellent high-voltage performance. Notably, with low-voltage cathode materials such as perylene-3,4,9,10-tetracarboxylicdianhydride (PTCDA [43]) and P3-type K_0.45_Rb_0.05_Mn_0.85_Mg_0.15_O_2_ (KMO [44]), both LHCE and CHVE exhibit good electrochemical performance (Figs S14 and S15). These results highlight the compatibility of LHCE with low-voltage cathodes and the broad applicability of CHVE with both low/high-voltage cathodes, providing a new perspective on electrolyte design and electrode–electrolyte compatibility.

Transmission electron microscopy (TEM) was used to characterize the cathode electrolyte interface (CEI) that was formed on the KVO electrodes that were cycled in LHCE and CHVE (Fig. 4g). Clearly, the CEI on KVO electrodes that were cycled in LHCE reveals thin and uniform characteristics after 5 cycles, yet significant thickness increase is obtained after 20 cycles, probably due to the severe electrolyte decomposition under a high voltage. By contrast, the CEI on KVO electrodes that were cycled in CHVE exhibits thin and uniform characteristics after 5 and 20 cycles, confirming the formation of a stable interphase and effectively mitigating electrolyte decomposition and Al foil corrosion.

The elemental composition of the CEI on the KVO electrode was further studied by using X-ray photoelectron spectroscopy (XPS) as a function of the Ar^+^ sputtering time (Figs S16 and S17). Clearly, the F 1s XPS peak intensity of the CEI at different etching depths in CHVE is higher than that in LHCE (Fig. S16), which is further verified by using the F atomic ratios (Fig. S17a). Meanwhile, the F:C atomic ratio of the CEI in CHVE is higher than that in LHCE (Fig. S17b). Therefore, the CEI on the KVO electrode in CHVE reveals F-rich components compared with that in LHCE, which accounts for the large aggregate clusters in CHVE that promote anion-dominated interfacial chemistry, further leading to enhanced electrochemical performance.

### Compatibility of cosolvent electrolyte with anodes and full-cells

It should be mentioned that CHVE not only exhibits excellent high-voltage cathode compatibility, but also delivers superior electrochemical performance with anodes, such as K||Cu and K||graphite. Under a current density of 0.25 mA cm^−2^ and areal capacity of 0.5 mAh cm^−2^, the K||Cu cells with CHVE exhibited excellent stability of 200 cycles with an average coulombic efficiency of 98.4% (Fig. S18a), exceeding those of the K||Cu cells in TE (10 cycles) and LHCE (135 cycles). Even under a high areal capacity of 1 mAh cm^−2^, the K||Cu cells with CHVE deliver an average coulombic efficiency of 98.8% within 130 cycles (Fig. S18b), significantly surpassing those in TE (1 cycle) and LHCE (25 cycles). Also, the Aurbach efficiency test of K||Cu cells (Fig. S19) further confirmed the advantages of using CHVE (98.8%), which is superior to TE (failed) and LHCE (97.8%). Obviously, CHVE exhibits superior performance, which is due to its unique solvation structure with large aggregate clusters. The plating/stripping morphology is further evaluated by using SEM after five cycles. Compared with the clean surface of pristine Cu foil (Fig. S20), the Cu foil with TE exhibits (i) a large amount of dead potassium after K-stripping and (ii) obvious dendritic growth and irregular morphology after K-plating (Fig. S21), which are consistent with its poor electrochemical performance. In sharp contrast, with LHCE (Fig. S22) and CHVE (Fig. S23), the surface of the Cu foil is similar to the pristine state without obvious dead potassium after K-stripping. The fact that dense and flat surface characteristics can be obtained after K-plating demonstrates that both LHCE and CHVE can reversibly support good K-plating/stripping.

Graphite is an ideal anode material for PIBs because of its low-voltage platform and environmentally friendly advantages, so it is important to evaluate its compatibility with the electrolytes that were used in this study. The K||graphite cell with TE reveals a high-voltage platform and a low reversible capacity (Fig. S24), corresponding to [K^+^–solvent]^+^ co-intercalation behavior. By contrast, under CHVE or LHCE, the K||graphite cells exhibit a low-voltage platform and high reversible capacity, which is directly related to the K^+^ intercalation behavior, validated by using the *insitu* X-ray diffraction experiments (Fig. S25). The K||graphite cells with TE and LHCE suffered from rapid capacity decay within 30 and 300 cycles, respectively. Remarkably, the K||graphite cells with CHVE delivered a reversible capacity of 230 mAh g^−1^ after 1200 cycles at a current density of 50 mA g^−1^, corresponding to a running time of >17 months—a record for cycling time in ether-based electrolyte systems (Fig. S26 and Table S6) [7,9,45–48]. Even under high mass-loading conditions, the K||graphite cells with CHVE still can maintain high reversible capacity and stably operate for >3 months (Fig. S27). These results unequivocally demonstrate the excellent compatibility between the CHVE and graphite anode, which is due to the formation of efficient SEI on the graphite electrode (Fig. S28).

The SEI morphologies on the graphite surface under various electrolytes were further investigated by using TEM experiments. An uneven and thick SEI was observed on the surface of graphite cycled with TE, while a comparatively thinner yet slightly uneven SEI formed when cycled with LHCE (Fig. S28). With CHVE, the graphite electrode exhibits a thin and homogeneous SEI, in line with its outstanding long-term cycling performance. The SEI compositions on the graphite surface were investigated by using XPS experiments. Although a slightly high content of F element was obtained in TE (Figs S29 and S30), it should be ascribed to the [K–solvent]^+^ co-intercalation behavior and the resulting severe electrolyte decomposition, which are consistent with the poor coulombic efficiency of the K||graphite cell. Compared with LHCE, a higher F content is observed in CHVE, indicating the presence of large aggregate clusters that facilitate the formation of anion-derived F-rich SEI (Figs S29 and S30). Additionally, the higher F:C atomic ratio further supports the formation of an anion-derived F-rich SEI on the graphite electrode that was cycled in CHVE (Fig. S30c). In summary, the large aggregate clusters in CHVE promote the formation of an anion-derived F-rich SEI on the graphite electrode, remarkably strengthening the cycling stability.

**Figure 5.**
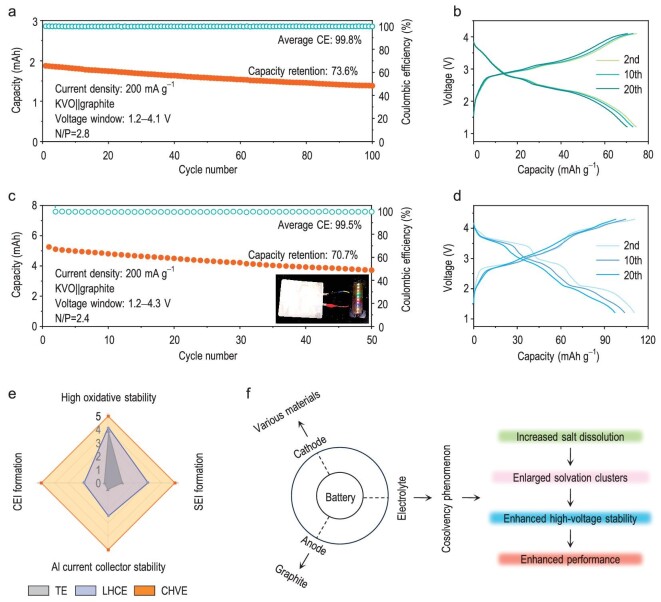
Electrochemical performance of KVO||graphite pouch full-cells. (a) Cycling performance and (b) charge–discharge curves of the KVO||graphite pouch-cell with a voltage range of 1.2–4.1 V. (c) Cycling performance and (d) charge–discharge curves of the KVO||graphite pouch-cell with a voltage range of 1.2–4.3 V. (e) Radar plot evaluating the three electrolytes. (f) Highlights the advantages of electrolyte induced by the cosolvency phenomenon.

To further explore the practical application prospects of CHVE in PIBs, pouch cells with KVO as the cathode and graphite as the anode were assembled and evaluated under different upper cut-off voltages. In the range of 1.2–4.1 V, the KVO||graphite pouch-cell (N/P = 2.8) exhibits a stable cycling capability of 100 cycles with an average coulombic efficiency of ≤99.8% (Fig. 5a). More significantly, the KVO||graphite pouch-cell exhibits satisfactory overlap of charge–discharge curves, directly reflecting its good cycling reversibility (Fig. 5b). In the increased range of 1.2–4.3 V, the KVO||graphite pouch-cell (N/P = 2.4) reveals a good average coulombic efficiency of 99.5% within 50 cycles (Fig. 5c) and displays a higher capacity and good charge–discharge platform (Fig. 5d). The inset in Fig. 5c shows a KVO||graphite pouch-cell lighting a LED panel, which intuitively proves the feasibility and efficiency in certain applications. Moreover, the CHVE also exhibits good compatibility with low-voltage full-cells, whose cathode is PTCDA or KMO and anode is graphite (Figs S31 and S32), further confirming the broad compatibility of CHVE with different cathode materials.

Our experimental results indicate that CHVE performs exceptionally well to passivate an Al current collector, enhance oxidation stability and form robust SEI/CEI (Fig. 5e). It also demonstrates outstanding compatibility with a range of cathode and anode materials (Fig. 5f). As an essential component of a battery system, CHVE increases the solubility of salts with the presence of a cosolvent, hence encouraging the efficient involvement of a greater number of anions in coordination that leads to large aggregate clusters. This not only increases the high-voltage stability, but also greatly boosts the overall performance of the battery. Our cosolvent electrolyte design strategy provides new avenues for the development of high-performance potassium-ion electrolytes and beyond.

### Extension to broader electrolyte systems

**Figure 6.**
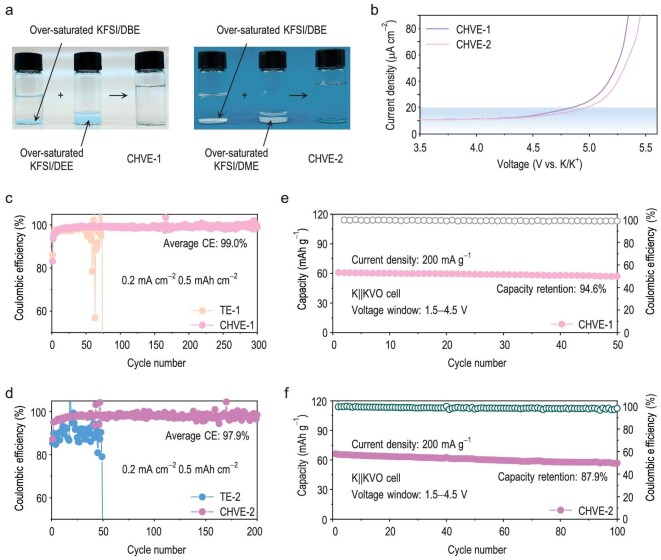
Electrochemical performance of broader electrolyte systems. (a) Optical photo of the presence of cosolvency phenomenon in DEE/DME and DBE mixtures. (b) LSV profiles of CHVE-1 and CHVE-2. Coulombic efficiency of K||Cu cells under a current density of 0.2 mA cm^−2^ and an area capacity of 0.5 mAh cm^−2^ in (c) CHVE-1 and (d) CHVE-2. K||KVO cycle performance in (e) CHVE-1 and (f) CHVE-2.

As described above, the cosolvency phenomenon as an effective strategy for regulating solvation structure and optimizing electrochemical performance has shown great potential for the design of high-performance electrolytes. In order to confirm the universality of the strategy, we expanded its utility to common ether-based solvents including 1,2-diethoxyethane (DEE) and 1,2-dimethoxyethane (DME), whose high-voltage stability is limited (Fig. S33). As expected, with the DBE cosolvent, the oversaturated KFSI/DEE electrolyte (and oversaturated KFSI/DBE electrolyte) forms a clear and transparent mixed electrolyte that is similar to the mixture of the oversaturated KFSI/DME (and oversaturated KFSI/DBE) electrolyte (Fig. 6a). This behavior demonstrates the broad feasibility of the cosolvent electrolyte design strategy. The two new cosolvent electrolytes, namely CHVE-1 (1.5 M KFSI in DEE–DBE) and CHVE-2 (1.5 M KFSI in DME–DBE), were further evaluated for battery performance. Both proved to be superior to the corresponding single-solvent electrolytes TE-1 (1.5 M KFSI in DEE) and TE-2 (1.5 M KFSI in DME). Table S7 contains the specific electrolyte preparation information. The LSV results (Fig. 6b) reveal the remarkably enhanced oxidation stability of CHVE-1 (5.0 V) and CHVE-2 (4.8 V), further confirming the positive influence of the cosolvent on high-voltage stability.

The K||Cu cells were used to evaluate the advantages of CHVE-1 and CHVE-2 over TE-1 and TE-2. Compared with TE-1 and TE-2, CHVE-1 and CHVE-2 significantly improved the K metal plating/stripping efficiency, with average coulombic efficiencies reaching 99.0% and 97.9%, respectively (Fig. 6c and d). Also, under a voltage range of 1.5–4.5 V, the K||KVO cell reveals a capacity retention of 94.6% after 50 cycles in CHVE-1 (Fig. 6e) and delivers a capacity retention of 87.9% after 100 cycles in CHVE-2 (Fig. 6f). Conclusively, the cosolvent electrolyte design strategy is a universal strategy and the corresponding cosolvent electrolytes exhibit noteworthy benefits in terms of enlarging anion-rich aggregate clusters, further leading to significantly enhanced electrochemical properties.

## CONCLUSIONS

In summary, we systematically proposed a cosolvent electrolyte design strategy for high-voltage and stable PIBs. Notably, by cleverly utilizing the cosolvency phenomenon, we significantly increased the solubility of the salt in the cosolvent electrolyte, promoting the participation of anions and enlarging the aggregate solvation cluster size. The enlarged aggregate solvation clusters further led to the formation of an effective electrode–electrolyte interphase and consequently enhanced the electrochemical performance of the battery. These key findings have been proven through synchrotron X-ray scattering experiments and theoretical simulation methods. Our cosolvent electrolyte design strategy provides a new perspective for electrolyte design, opening up new avenues for the development of high-voltage PIBs and beyond, which is expected to promote continuous innovation and progress in battery technology.

## Supplementary Material

nwae359_Supplemental_File
