# Supplementary material for: Cosolvent electrolyte chemistries for high-voltage potassium-ion battery
Source: Natl Sci Rev. 2024 Oct 15;11(11):nwae359. doi: 10.1093/nsr/nwae359 (PMC11533897; doi:10.1093/nsr/nwae359)
Supplement: nwae359_Supplemental_File [file nwae359_supplemental_file.pdf]

Supplementary Data

Cosolvent electrolyte chemistries for high-voltage potassium-ion battery

Mengkang Shen[1#], Zhongqin Dai[2#], Ling Fan[1*], Hongwei Fu[1], Yuanhui Geng[1], Jie Guan[1], Fanfei Sun[3,4*], Apparao M. Rao[5], Jiang Zhou[6], Bingan Lu[1,7*]

[1]School of Physics and Electronics, Hunan University, Changsha 410082, P. R. China

[2]School of Physical Science and Technology, Shanghai Tech University, Shanghai, 201210 P. R. China

[3]Shanghai Synchrotron Radiation Facility, Shanghai Advanced Research Institute, Chinese Academy of Sciences, Shanghai 201204, China

[4]Shanghai Institute of Applied Physics, Chinese Academy of Sciences, Shanghai 201204, China

[5]Department of Physics and Astronomy, Clemson Nanomaterials Institute, Clemson University, Clemson, SC, 29634, USA

[6]School of Materials Science and Engineering, Central South University, Changsha 410083, P. R. China

[7]State Key Laboratory of Advanced Design and Manufacturing for Vehicle Body, Hunan University, Changsha 410082, China

[*]Corresponding author: Ling Fan (fanling@hnu.edu.cn); Fanfei Sun (sunff@sari.ac.cn); Bingan Lu (luba2012@hnu.edu.cn)

[#]These authors contributed equally to this work.

METHODS

Electrolytes and materials preparation

All electrolytes were prepared and processed in a glovebox filled with argon gas, with concentrations of O_2 and H_2O both less than 0.01 ppm. Potassium bis(fluorosulfonyl)imide (KFSI, >99%) was used as the salt. Diglyme (DGM, >99%), 1,2-dibutoxyethane (DBE, >99%), 1,1,2,2-Tetrafluoroethyl-2,2,3,3-tetrafluoropropylether (TTE, >98%), 1,2-dimethoxyethane (DME, >99%), 1,2-diethoxyethane (DEE, >99%) were used as solvents. TE were prepared by dissolving 1.5 mmol KFSI in 1.0 ml DGM solvent. Similar techniques available for other electrolyte preparation processes. Prussian blue (PB), δ-$K_{0.51}V_2O_5$ (KVO) and P3-type $K_{0.45}Rb_{0.05}Mn_{0.85}Mg_{0.15}O_2$ (KMO) were synthesized based on existing literatures[1-3]. The perylene-3,4,9,10-tetracarboxylicdianhydride (PTCDA) was purchased from Energy Chemical and prepared as previous literature[4].

Electrodes preparation

The graphite electrode was prepared by mixing the graphite: Super P: CMC binder at a ratio of 8:1:1 in H_2O: absolute alcohol solutions to form a uniform slurry, then the slurry was coated on Cu foil and dried at 80 °C drying cabinet for at least 12h. The PB and KVO electrodes were prepared by mixing corresponding active materials with Super P and PVDF binder in NMP in a weight ratio of 6:3:1. The preparation method for KMO and PTCDA is the same as the above cathode electrodes, only changing the mass ratio to 7:2:1. All of the cathode materials were coated on carbon-coated Al foil and dried at 80°C overnight under vacuum. The average mass loadings of graphite electrode, PB electrode, KVO electrode, PTCDA electrode and KMO electrode are around 1.0-2.0 mg cm^{-2}, 0.8-2.0 mg cm^{-2}, 1.0-1.8 mg cm^{-2}, 1.2-1.8 mg cm^{-2}, 1.0-2.5 mg cm^{-2}, respectively. The high mass loading graphite electrodes is approximately between 5-10 mg cm^{-2}.

Electrochemical performance testing

The 2032-type coin-cells were assembled and used to electrochemical performance investigation. The K||graphite, K||PB, K||KVO, K||PTCDA, K||KMO, K||Cu and K||Al half-cells were assembled using K foil as the counter/reference electrode, Whatman glass fiber as separator and the prepared cathode/anode as work electrodes. For full-cells, the graphite electrode should take a pre-potassium treatment, it was matched with the corresponding cathode electrode materials to assemble pouch-

cells. The capacity ratio of negative to positive electrodes is around 2.4. The electrolyte dosage is 80 μL for each coin-cell. Appropriate charge-discharge voltage ranges were used for K||graphite (0.01–3 V), K||PB (2.0–4.5 V), K||KVO (1.5–4.5 V), K||PTCDA (1.5–3.5 V), K||KMO (1.5–3.9 V) cells. The K||Cu cells were tested by a repeated step of plating K (0.5/1.0 mAh cm^{-2}) on Cu foil, followed by a stripping process (charge to 1.0 V) under a current density of 0.2/0.25 mA cm^{-2}. The Aurbach efficiency test was performed as follows: firstly, plating 2.5 mAh cm^{-2} K on Cu foil, and subsequently stripping until the voltage reached 1.0 V under a current density of 0.25 mA cm^{-2}. And then plating 2.5 mAh cm^{-2} K on Cu foil again, subsequently stripping/plating 0.5 mAh cm^{-2} K, repeat this process 10 times, and finally charge to 1.0 V under a current density of 0.25 mA cm^{-2}. The LSV was performed using K||Al cells under the voltage range of 2.0–6.0 V and a scan rate of 1 mV s^{-1}.

Electrode and interphase characterization

Transmission electron microscope (FEI Titan G2 60-300, 200kV) and scanning electron microscope (Jeol, JSM-7610Fplus) were used to analyze the electrode structure and morphology. The anodes/cathodes after cycling need to be washed by pure DME solvent in the glove box before send to characterize. XPS spectra were performed with a monochromatic Al Kα X-rays to investigate the chemical compositions of CEI/SEI. Raman (Renishaw2000, 785 nm laser excitation) was used to examine the solvation structure of electrolytes.

Theoretical calculations

Complexation energies were calculated using Gaussian 16 C0.1 under B3LYP exchange-correlation functional with Grimme's DFT-D3(BJ) empirical dispersion correction. Basis set of def-TZVP was adopted for structural optimizations, and ma-TZVP was used in single point energy calculations. We corrected the basis set superposition error with the counterpoise method.

MD simulations were carried out using the LAMMPS package[5]. The OPLS-AA force field[6] with fitted parameters for K^+ cations, FSI^- anions[7], DGM, DBE and TTE molecules (from LigParGen)[8-10] were used in this work. Parameter fitting was performed using the density functional theory (DFT) package Gaussian16 C0.1[11] under B3LYP exchange-correlation functional with Grimme's DFT-D3(BJ) empirical dispersion correction[12,13], the ma-TZVP[14,15] basis set was adopted for self-consistent field (SCF) calculations. The RESP2 (0.5)[16] charges

combining gas- and liquid-phase (implicit solvent, PCM model) charges were adopted for FSI^- anions, DGM, TTE and DBE molecules in this work calculated using Gaussian16 C0.1 and Multiwfn package[17,18]. Care must be taken to include electronic polarization effects in MD simulations of low dielectric constant solvents when using non-polarizable all-atom force field. Thus we replaced the partial atomic charges on the ions (K^+ and FSI^-) by effective, rescaled, charges q_i^{eff}, according to[19,20]

$$q_i^{eff} = \frac{q_i}{\sqrt{\varepsilon_\infty}} \tag{1}$$

Here, ε_∞ is the high-frequency contribution to the solvent permittivity stemming from electronic fluctuations in the solvent molecules. It can be related to the refractive index n as $\varepsilon_\infty = n^2$.

The simulation box was composed of 105 KFSI molecules, 493 DGM for TE, 135 KFSI molecules, 253 DGM and 357 TTE for LHCE and 117 KFSI molecules, 91 DGM and 312 DBE for CHVE. The systems were initialized using the Packmol package[21] and the Moltemplate package[22]. The systems were then subjected to a simulated annealing equilibration protocol as follows: (1) equilibration at T = 298 K for 2 ns in NPT ensemble; (2) heating up the system to 500 K over 2 ns in NPT ensemble; (3) relaxation at T = 500 K over 2 ns in NPT ensemble; (4) cooling down to T = 298 K over 2 ns in NPT ensemble; (5) equilibration at T = 298 K for 50 ns in NPT ensemble. The production run was subsequently performed at T = 298 K over 50 ns in NVT ensemble. All the simulations in this work used a timestep of 2 fs, and a pressure of 1 atm. The temperature and pressure were regulated with a Nose-Hoover thermostat and barostat, with a damping parameter of 0.2 ps and 2 ps respectively. The LAMMPS was used to obtain coordinated numbers and radial distribution functions. An in-house Python script was used to determine the percentage of SSIPs, CIPs, and AGGs in the initial solvation sheath.

X-ray scattering characterization

Synchrotron WAXS measurements were conducted at beamline BL16B at Shanghai Synchrotron Radiation Facility (SSRF). A photon energy of 10 keV (focusing spot size = 0.35 (h) × 0.58 (v) mm^2) was used for the experiment, and Pilatus 900k detectors were used to gather WAXS signals. The detector has 172 μm × 172 μm pixels. Silver behenate is used to calibrate the distance between the WAXS sample and the detector. Additionally, the standard sample was placed in the same location as the experimental samples and sealed in Kapton sample cells. A peristaltic pump was used in the

in-situ experiment to constantly pump the reaction mixture at a speed of 1.5 mL min^{-1}, while another peristaltic pump was used to inject the DBE solvent slowly. Prior to usage, both peristaltic pumps underwent meticulous calibration. Every two minutes, in-situ WAXS patterns were recorded, with a 20 s exposure period for each frame. After integrating the gathered 2D WAXS patterns into 1D scattering curves with Fit 2D software, the background signal from the solvent and Kapton sample cells was removed[23,24].

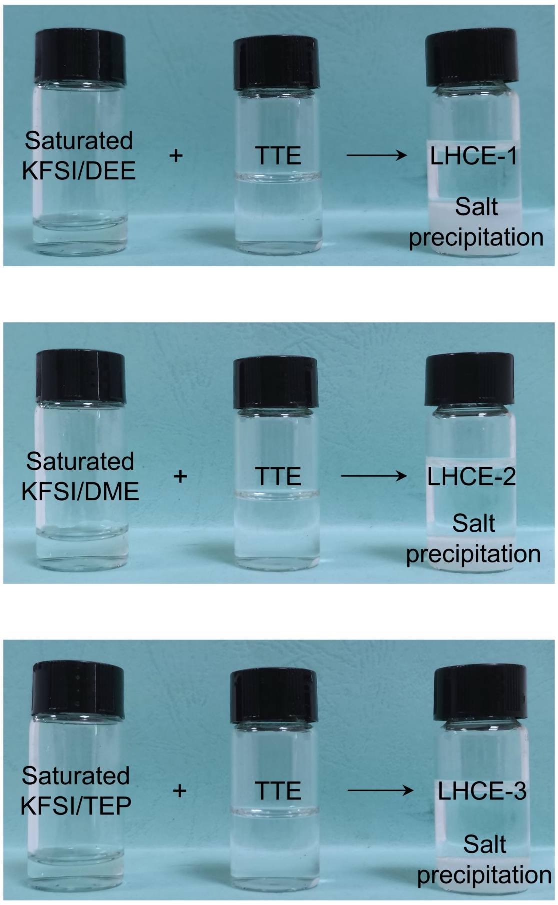

Fig. S1 Some examples of salt precipitation phenomenon in potassium-based electrolytes. Dilution of saturated KFSI/DME, KFSI/DEE, and KFSI/TEP electrolytes with TTE solvent.

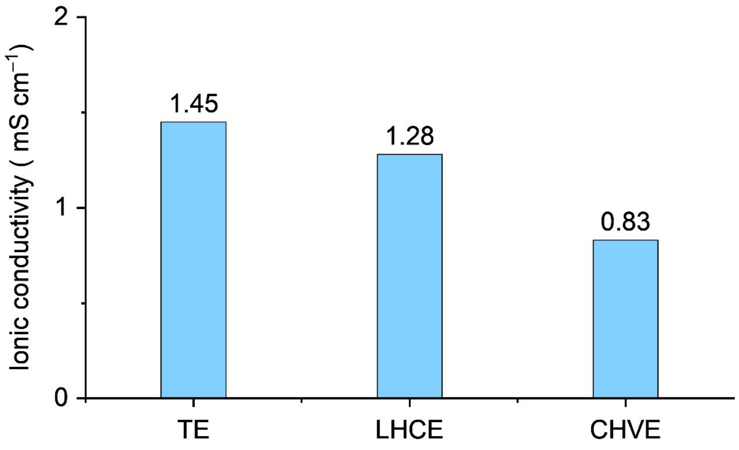

Fig. S2 Ionic conductivity of the three electrolytes (TE, LHCE and CHVE) used in this study.

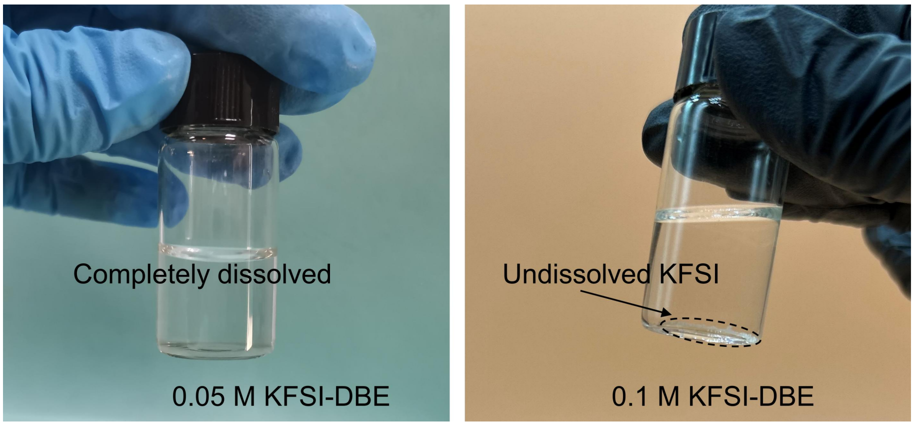

Fig. S3 Photograph of 0.05M KFSI and 0.1M KFSI in DBE.

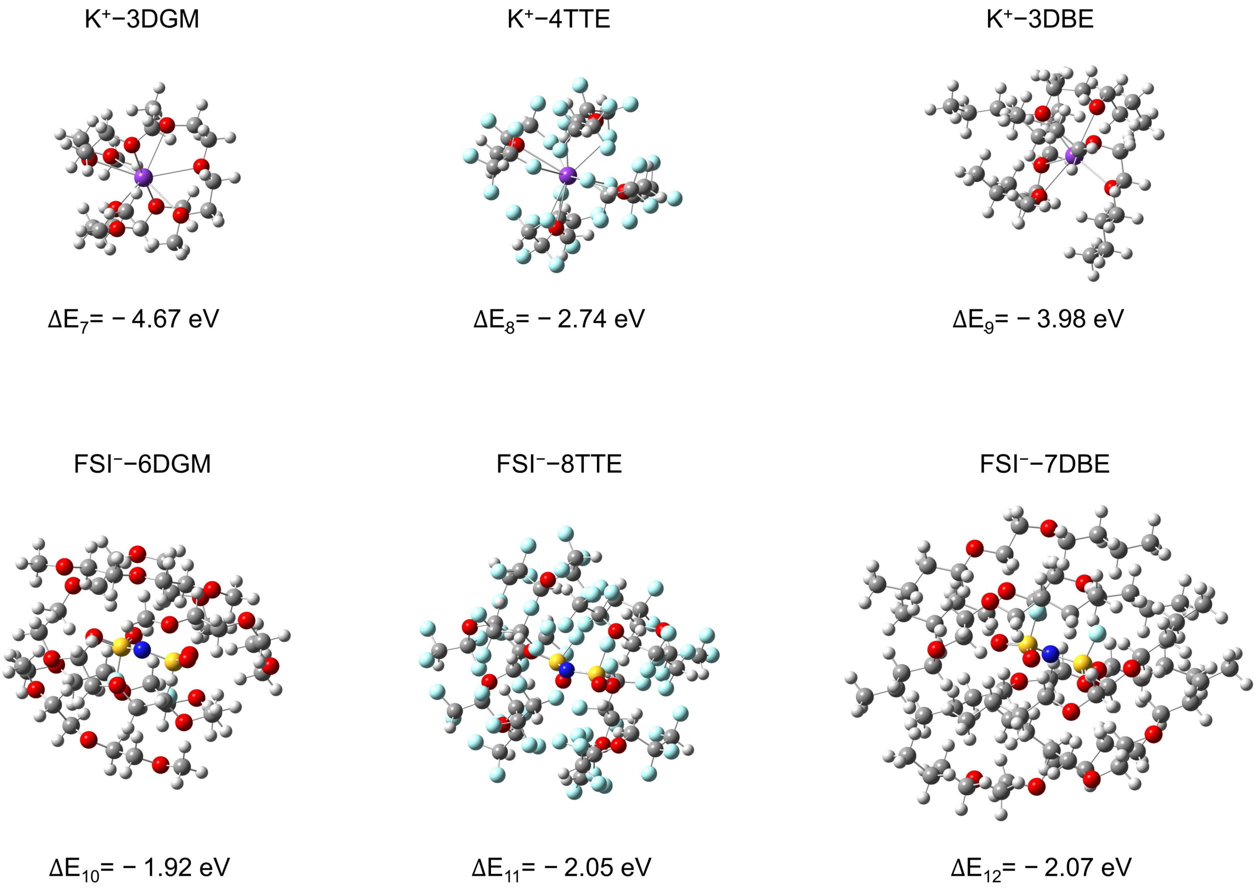

Fig. S4 The binding energies between K^+ (or FSI^-) with DGM, TTE and DBE solvent molecules, respectively (The solvent completely surrounds the K^+ or FSI^-).

Note: To eliminate the influence of solvent molecule size on interactions, we further calculated the binding energy when K^+ (or FSI^-) is completely surrounded by solvent molecules. The results indicate that for K^+, it still has the strongest binding energy with DGM. The binding energy between TTE and K^+ is the weakest (and from the calculated solvation environment, it is almost impossible for K^+ to be completely surrounded by TTE), which corresponds to the result that TTE can hardly dissolve KFSI. For FSI^-, the interaction between DGM and FSI^- is the weakest, while the interaction between DBE and FSI^- is the strongest. This further indicates that DBE has a strong interaction with FSI^- and can bind more effectively with anions. Therefore, the mixed solution of DGM and DBE can effectively improve the solubility of KFSI at appropriate ratios.

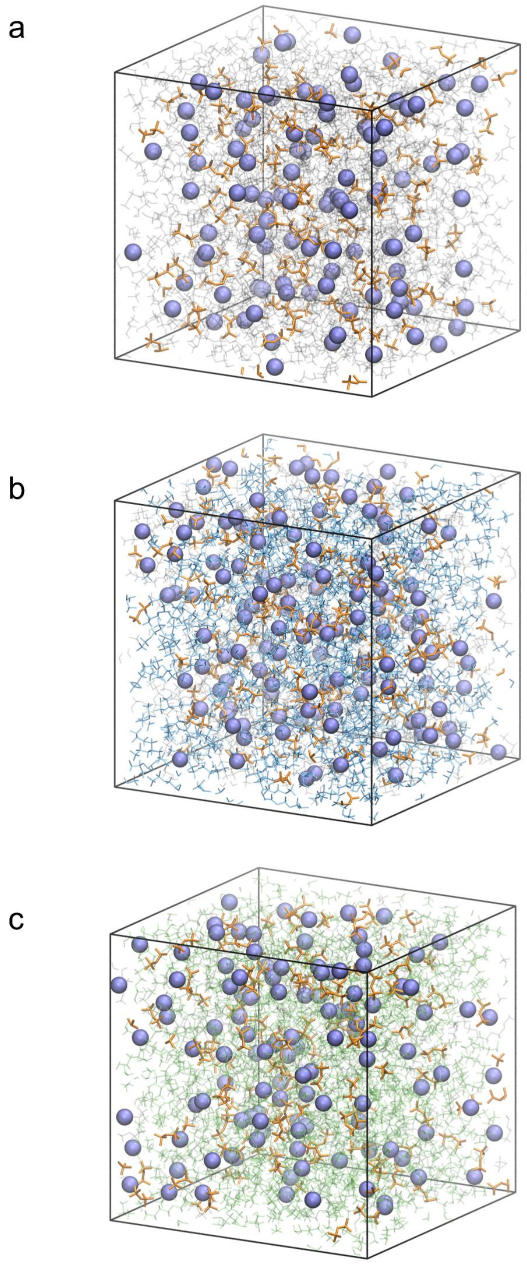

Fig. S5 Snapshot of K^+ solvation environments in the three electrolytes (a) TE, (b) LHCE and (c) CHVE.

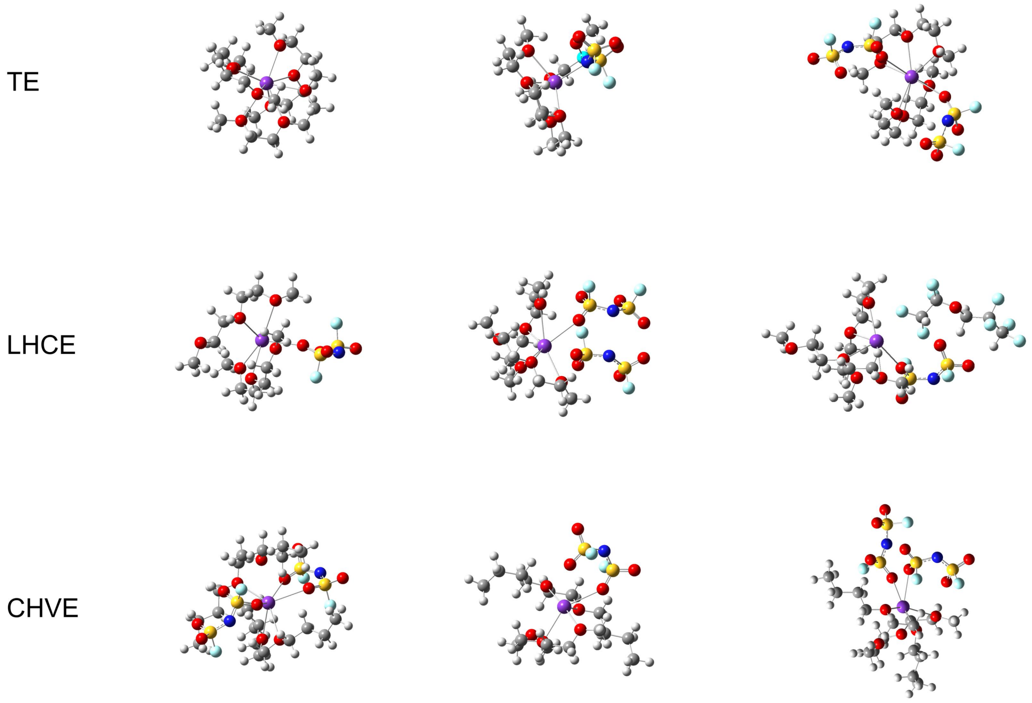

Fig. S6 The main solvation structure of K^+ was obtained by molecular dynamics simulation.

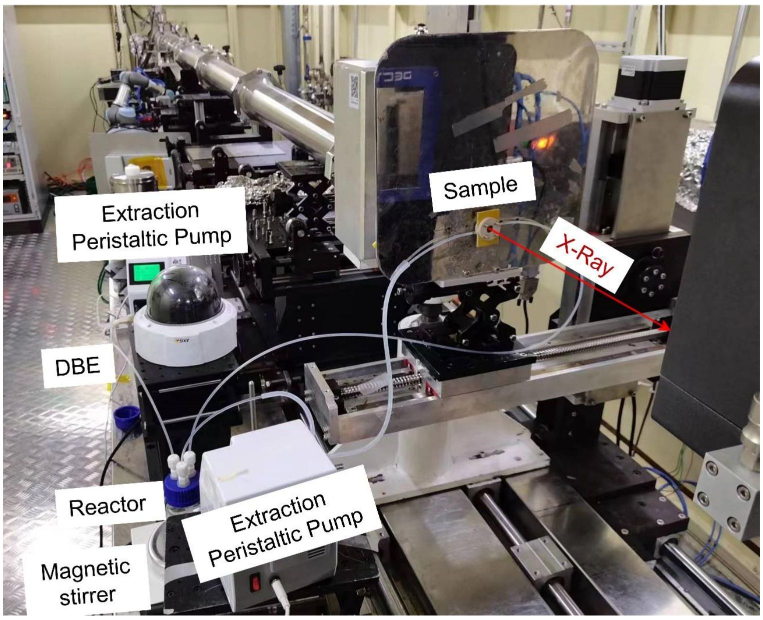

Fig. S7 Photograph of the *in-situ* WAXS experimental setup.

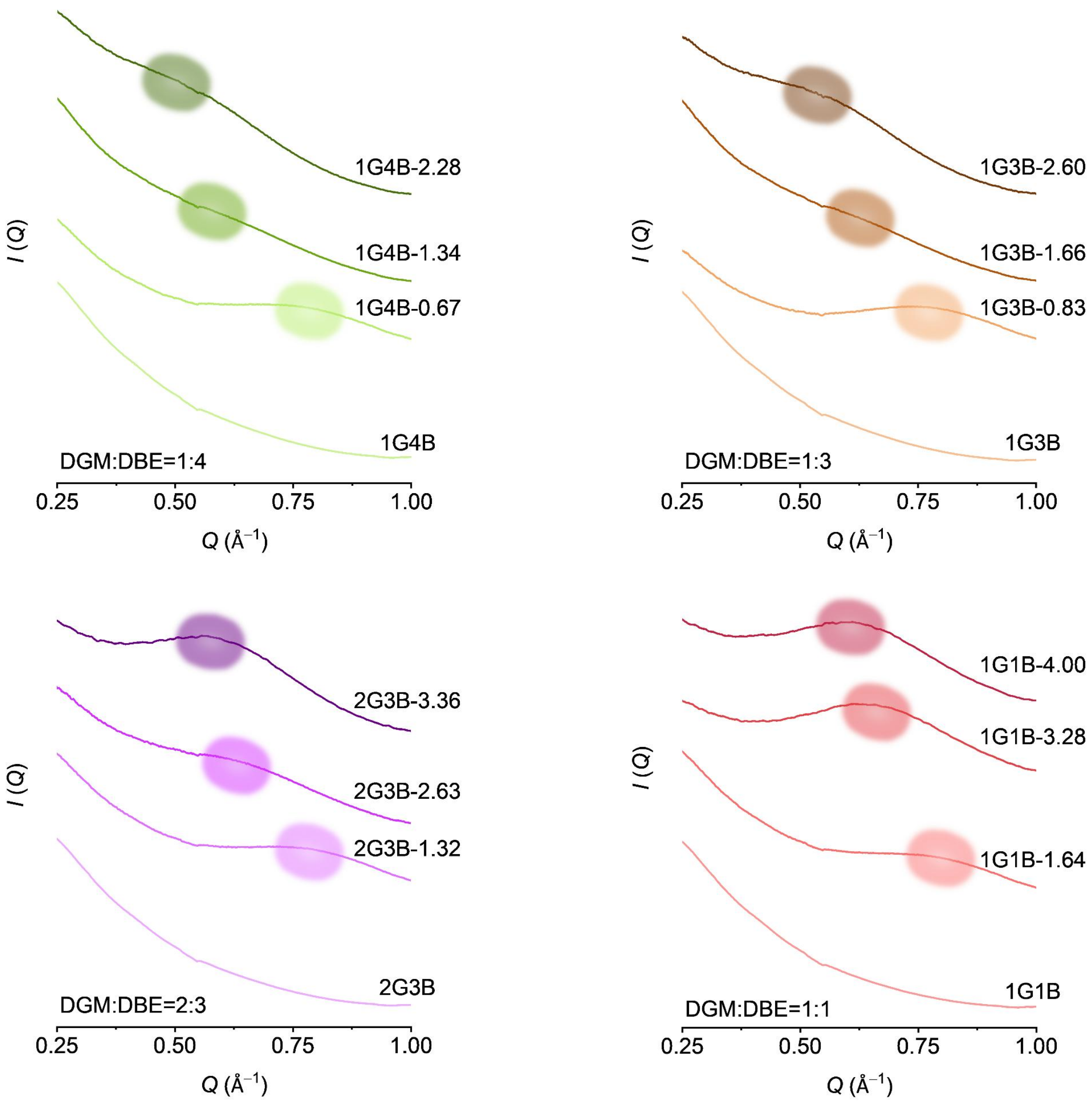

Fig. S8 The variation of WAXS curves with increasing KFSI concentration under different DGM:DBE ratios. The trialing numbers in each legend that labels each curve represents the moles of soluble KFSI.

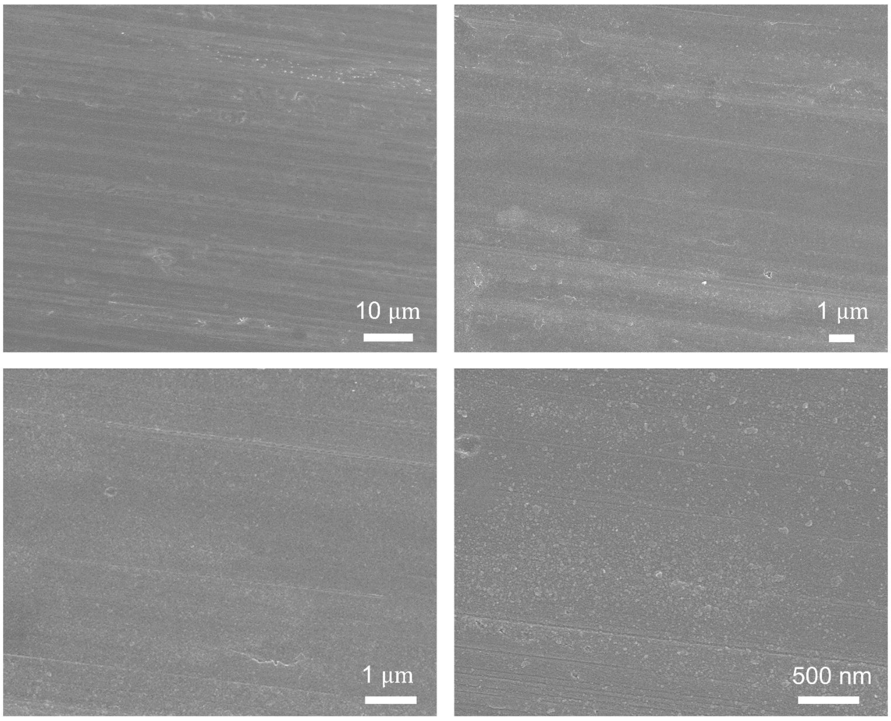

Fig. S9 The SEM images of pristine aluminum foil at different magnifications.

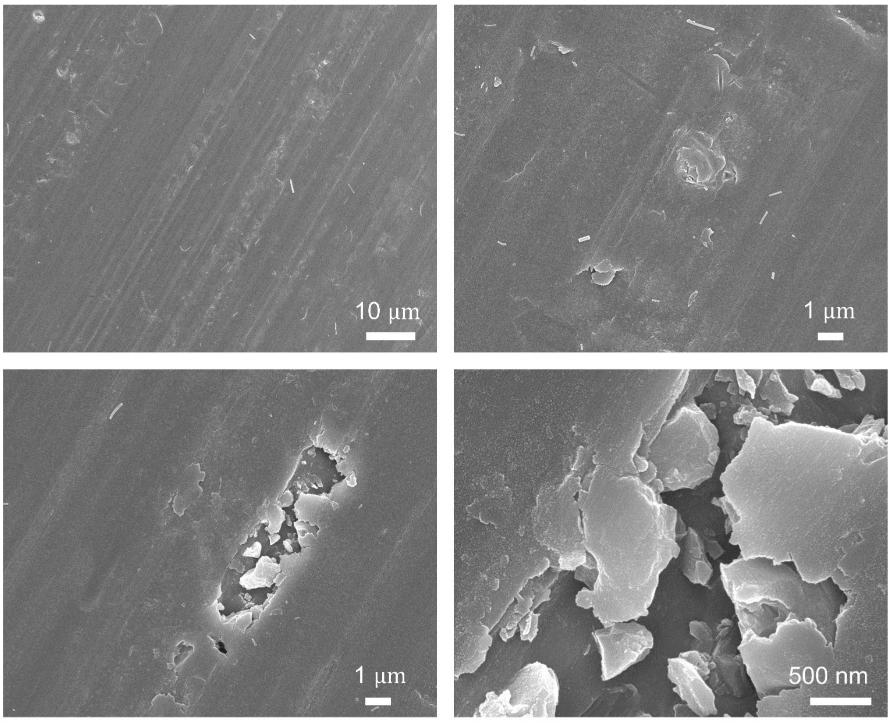

Fig. S10 The SEM images with different magnifications of aluminum foil corrosion caused by TE under high voltage conditions.

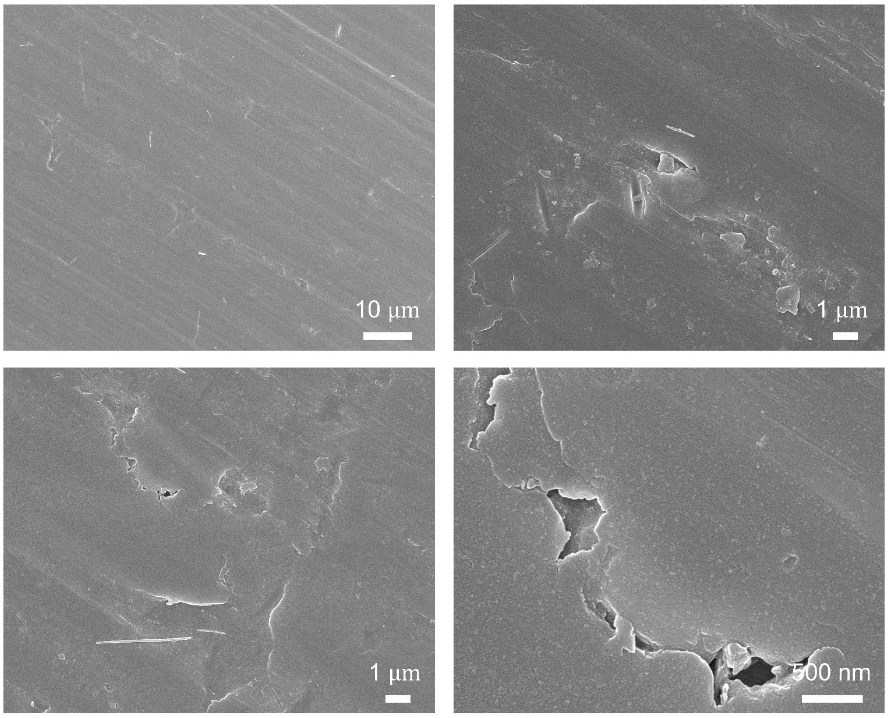

Fig. S11 The SEM images with different magnifications of aluminum foil corrosion caused by LHCE under high voltage conditions.

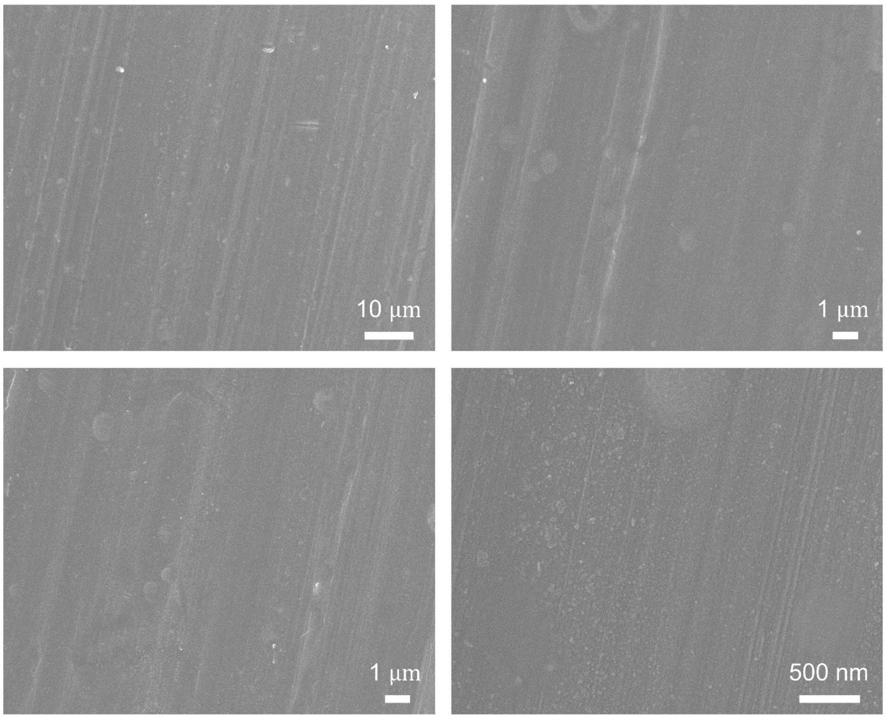

Fig. S12 The SEM images with different magnifications of aluminum foil with CHVE under high voltage conditions show the absence of corrosion.

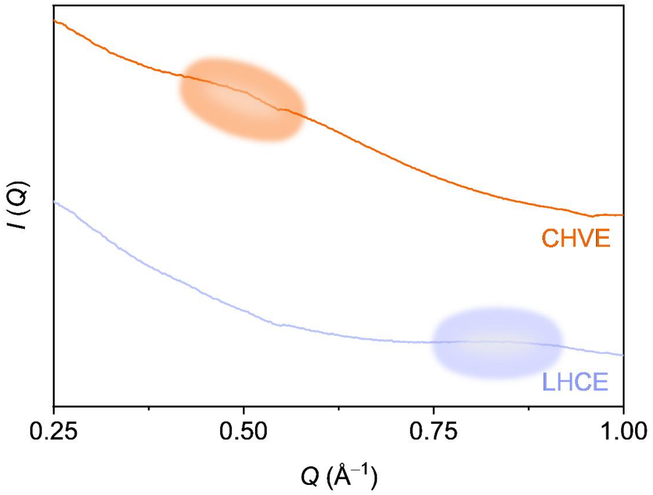

Fig. S13 The WAXS curves of LHCE and CHVE.

Note: In CHVE, the position of the characteristic peak observed by wide-angle X-ray scattering (WAXS) testing is approximately 0.48 Å^{-1}, and the average diameter of the corresponding cluster is estimated to be around 13.09 Å ($D=2\pi/Q$). This result clearly indicates the presence of significantly larger scale solvated cluster structures in the CHVE. In contrast, the WAXS characteristic peak is present around 0.88 Å^{-1} in LHCE, and the estimated cluster size is about 7.14 Å. This comparative WAXS data indicates that the solvation cluster structure in LHCE electrolyte is significantly smaller. The difference in the solvated cluster size is closely related to the solvation kinetics inside the electrolyte and the ion coordination environment.

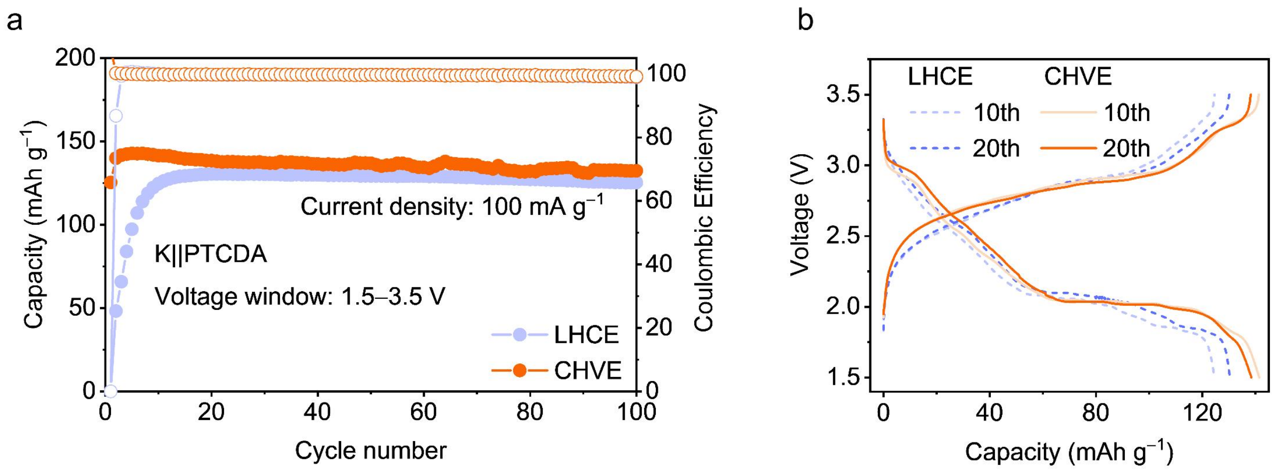

Fig. S14 PTCDA's cycle performance (a) and charge-discharge (b) characteristics with LHCE/CHVE when operated in the 1.5–3.5 V range.

Note: When the K||PTCDA cell is operated in the 1.5–3.5 V range using either the LHCE or CHVE, it exhibits excellent electrochemical performance. Specifically, the K||PTCDA cell operates stably for 100 cycles, and the reversible capacity with CHVE is slightly higher than that with LHCE. Furthermore, the charge-discharge curves with LHCE and CHVE exhibit a high degree of overlap, demonstrating the high reversibility of the K||PTCDA cell during the charge-discharge process.

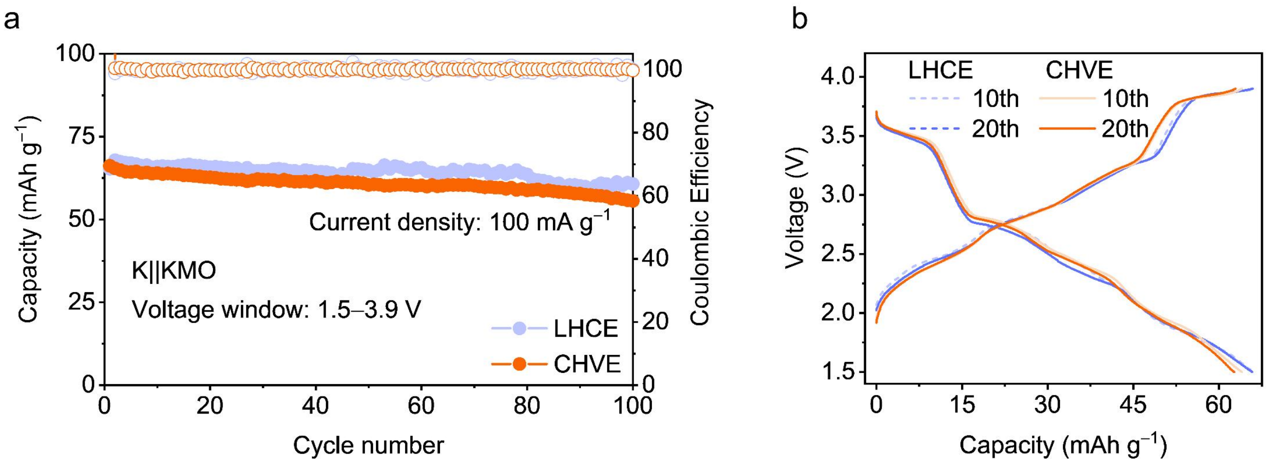

Fig. S15 KMO's cycle performance (a) and charge-discharge (b) characteristics with LHCE/CHVE when operated in the 1.5–3.9 V range.

Note: When the K||KMO cell is operated in the 1.5–3.9 V range using either LHCE or CHVE, the cell operates stably for 100 cycles. With LHCE, the cell exhibits slightly higher reversible capacity. The overlapping charge-discharge curves reflect good reversibility of the electrochemical reactions, and the cycling stability of the cell. The series of experimental results in this and the preceding figure validate the broad compatibility of LHCE and CHVE with low-voltage cathode materials.

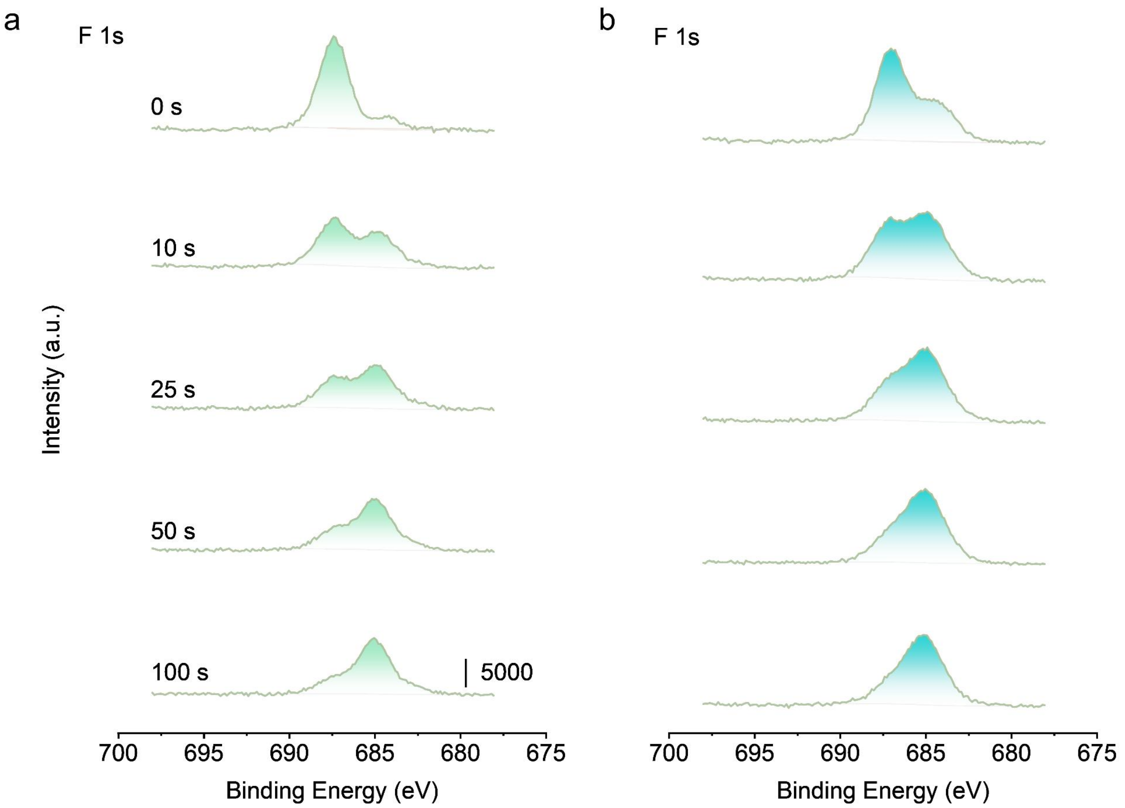

Fig. S16 F 1s X-ray photoelectron spectroscopy of the KVO electrode as a function of sputtering time after 20 cycles in (a) LHCE and (b) CHVE.

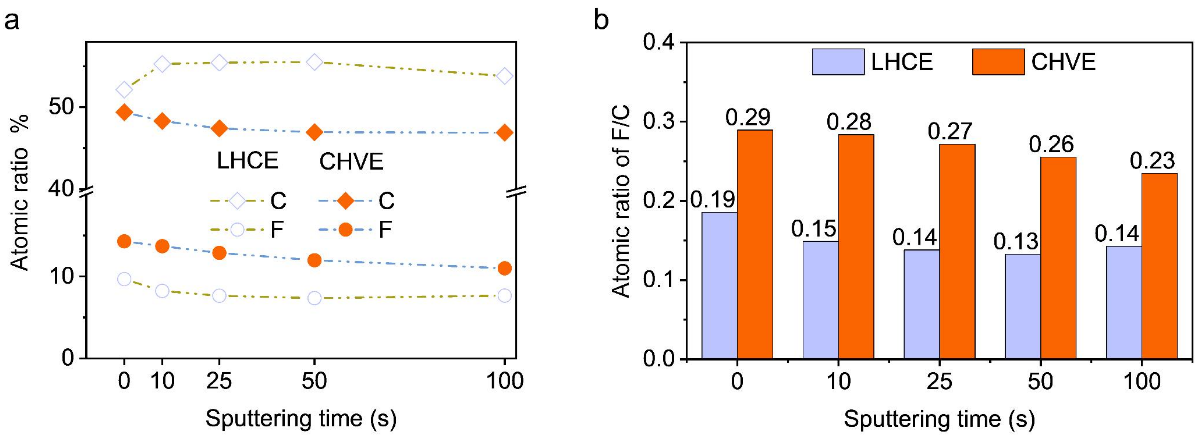

Fig. S17 (a) The atomic ratios of C and F elementals in KVO electrodes surface (after 20 cycles) at different depths. (b) The atomic ratios of F: C in KVO electrodes surface (after 20 cycles) at different depths.

Note: A more detailed and in-depth analysis quantified the atomic ratios of different elements present on the surface of the KVO electrode. With CHVE, the F element present before sputtering is 14.29%, which drop to 11% after 100 s of sputtering time (Fig. S17a). At the same time, the C content remains at a relatively low level (below 50%). Comparatively, with LHCE, the initial F content was only 9.68%, which further decreased to 7.67% after 100 s of sputtering time, while the C content fluctuated at a relatively high level (around 55%). In addition, the atomic ratio of F to C (F: C) further reveals the composition distribution of CEI. Compared with LHCE, CHVE consistently exhibits a higher F: C ratio, which directly indicates that the CEI in CHVE contains more abundant fluoride components. This is due to the involvement of more densely packed anions in CHVE solvation shell, which promotes the formation of larger AGGs, thereby determining the chemical composition and structural characteristics of CEI.

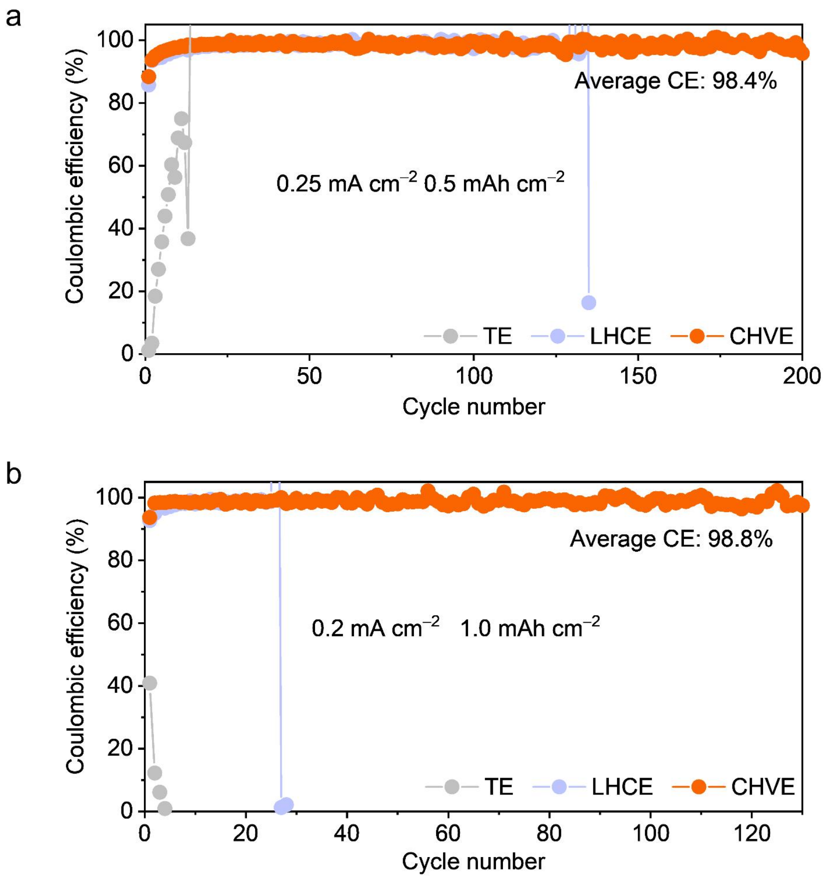

Fig. S18 Coulombic efficiency of K||Cu cells with different electrolytes. (a) Coulombic efficiency of K||Cu cells under a current density of 0.25 mA cm^{-2} and an area capacity of 0.5 mAh cm^{-2}. (b) Coulombic efficiency of K||Cu cells under a current density of 0.2 mA cm^{-2} and an area capacity of 1.0 mAh cm^{-2}.

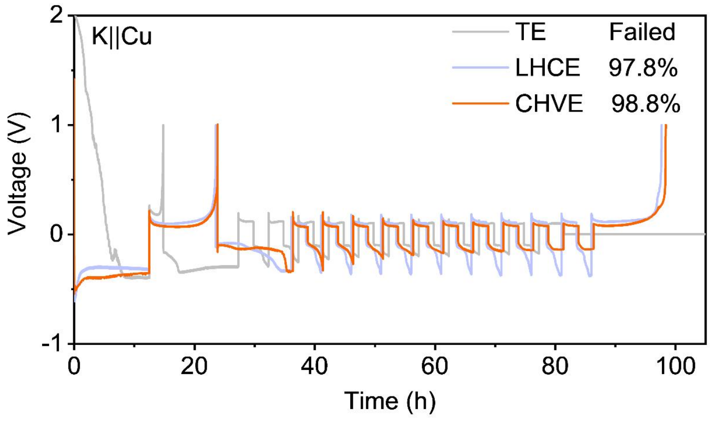

Fig. S19 Aurbach efficiency of the K||Cu cells in the three electrolytes.

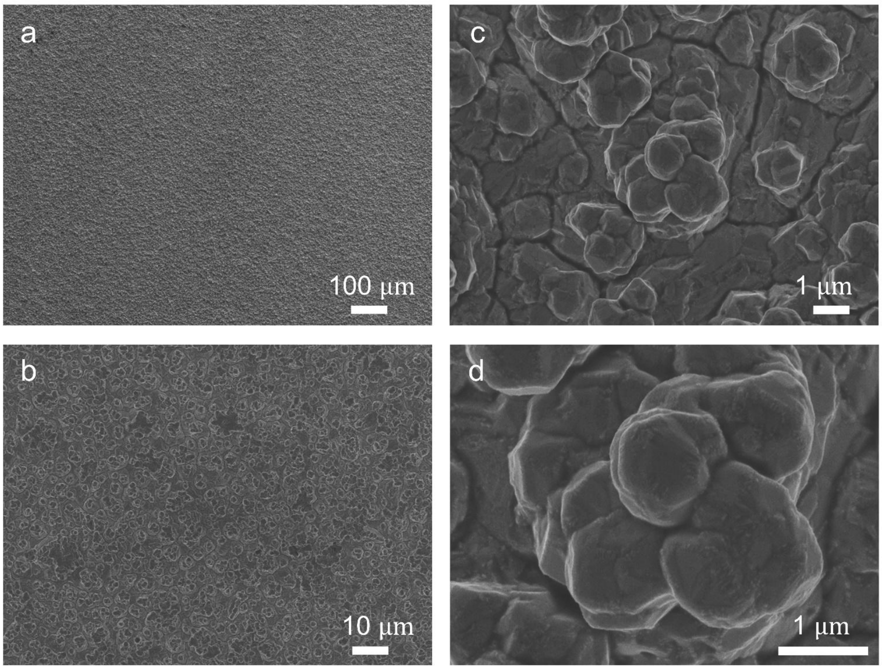

Fig. S20 The SEM images of the pristine Cu foil at different magnifications.

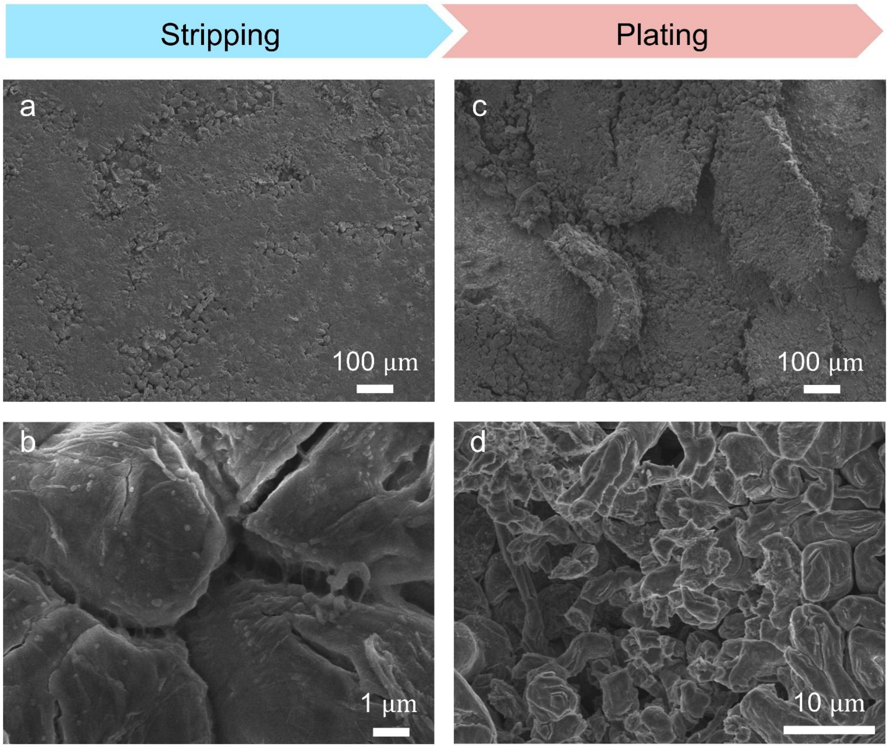

Fig. S21 The SEM images of Cu foil at different magnifications after 5 cycles in TE. (a-b) stripping K and (c-d) plating K metal.

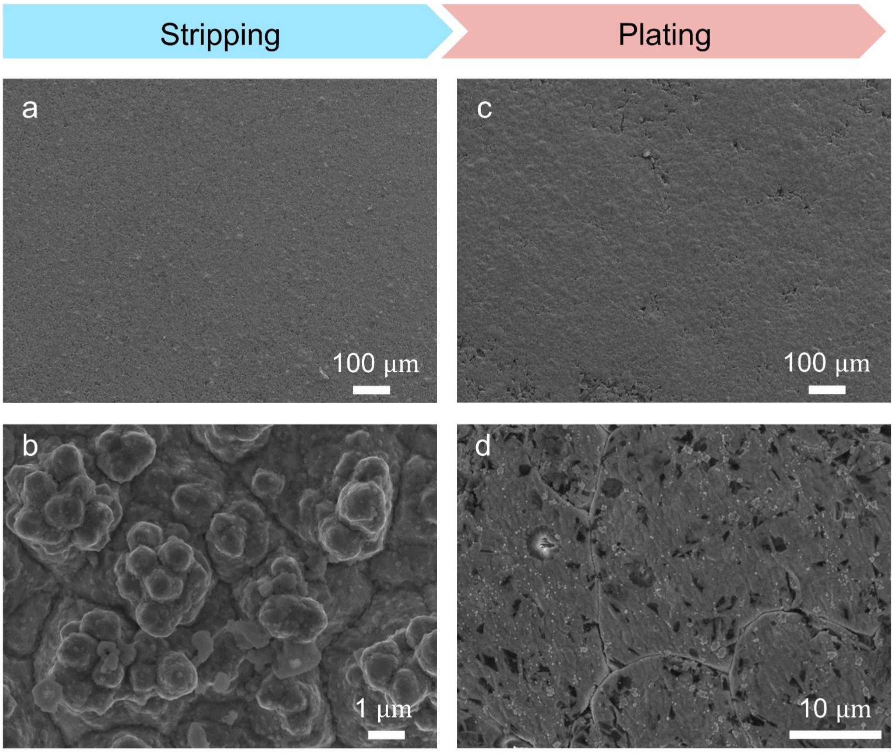

Fig. S22 The SEM images of Cu foil at different magnifications after 5 cycles of (a-b) stripping K and (c-d) plating K metal in LHCE.

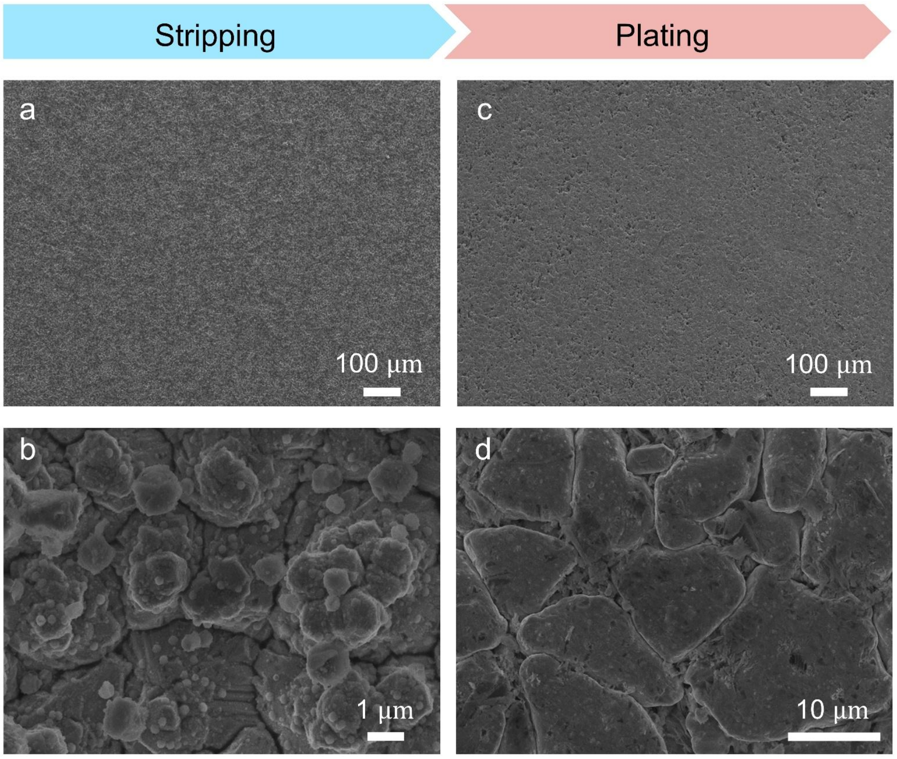

Fig. S23 The SEM images of Cu foil at different magnifications after 5 cycles in CHVE. (a-b) stripping K and (c-d) plating K metal.

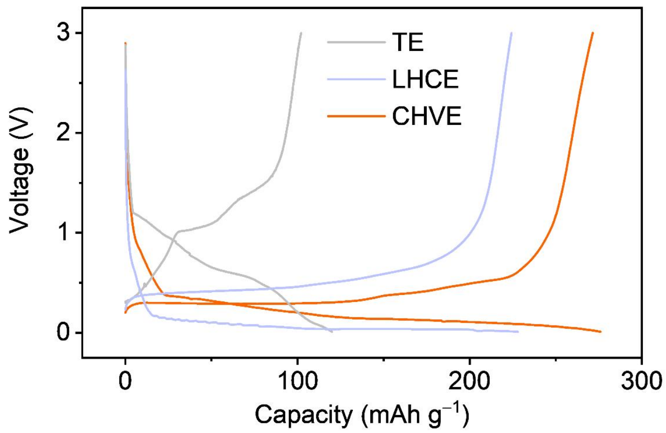

Fig. S24 Charge and discharge curves of K||graphite cells with various electrolytes of TE, LHCE or CHVE.

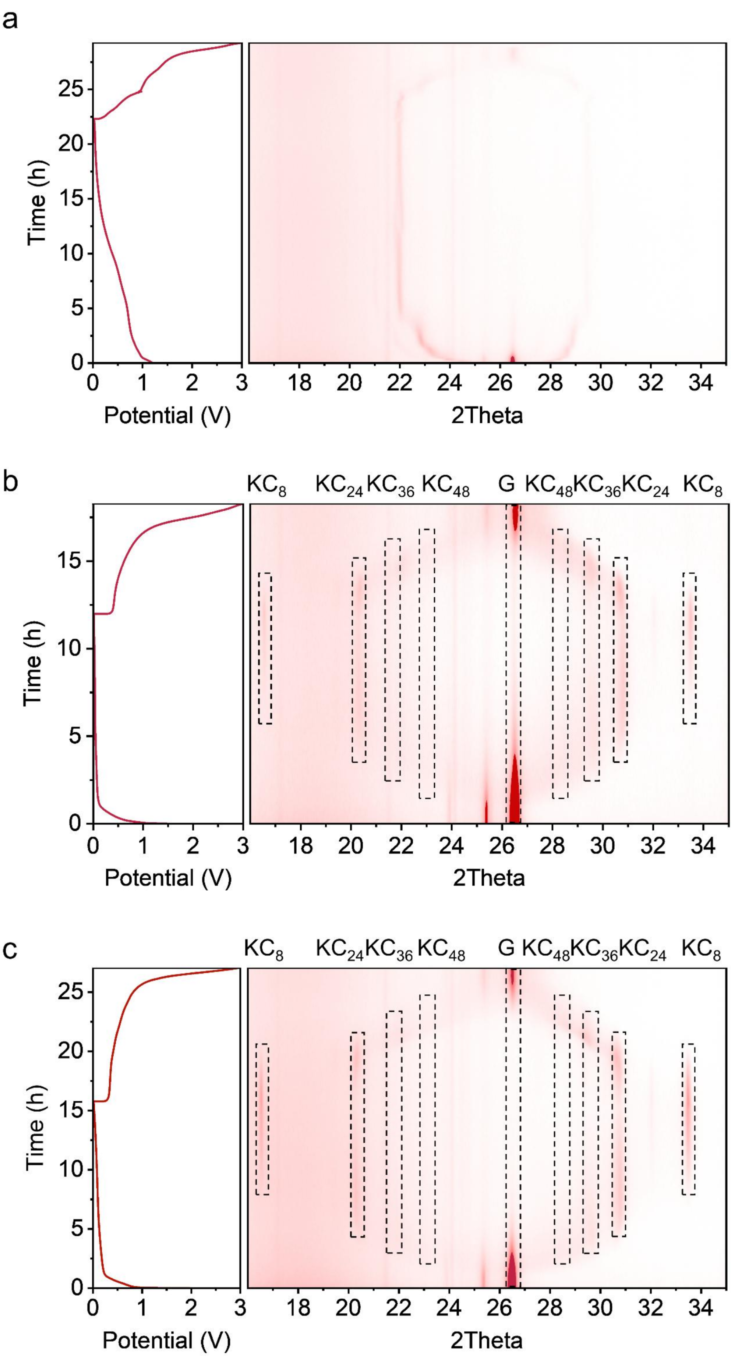

Fig. S25 *In-situ* X-ray diffraction contour plot of K||graphite cells in (a) TE, (b) LHCE and (c) CHVE during the initial cycle.

Note: With TE, the XRD spectra exhibit typical [K-solvent] $^+$ co-intercalation characteristics (Fig. S25a), which can be attributed to the strong interaction between K^+ and solvent molecules. With the LHCE or CHVE, the XRD spectra clearly demonstrate the reversible formation and disappearance of K-graphite intermediate phases such as KC_{48}, KC_{36}, KC_{24}, and KC_8, directly implying efficient reversible intercalation behavior of K^+ in graphite layers (Fig. S25b and c).

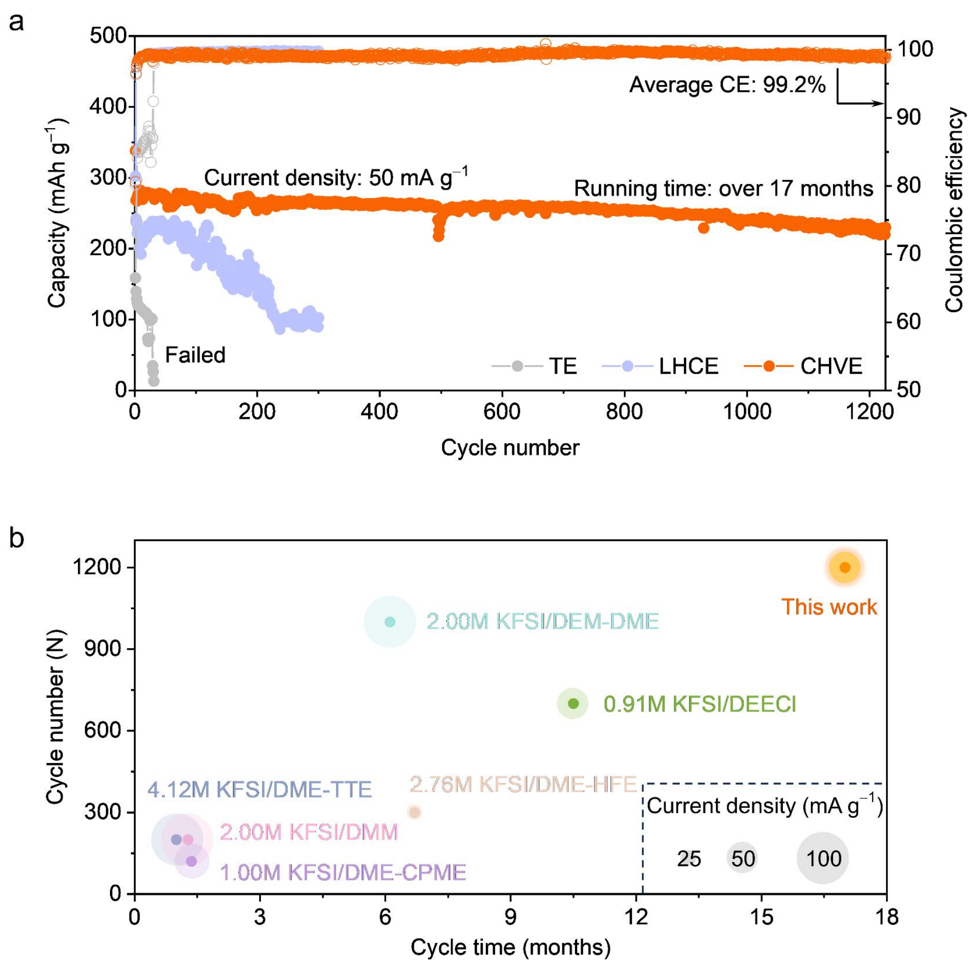

Fig. S26 (a) Long cycle performance of K||graphite in the three electrolytes used in this study. (b) Comparison of K||graphite cycle time and cycle number in different ether-based electrolytes.

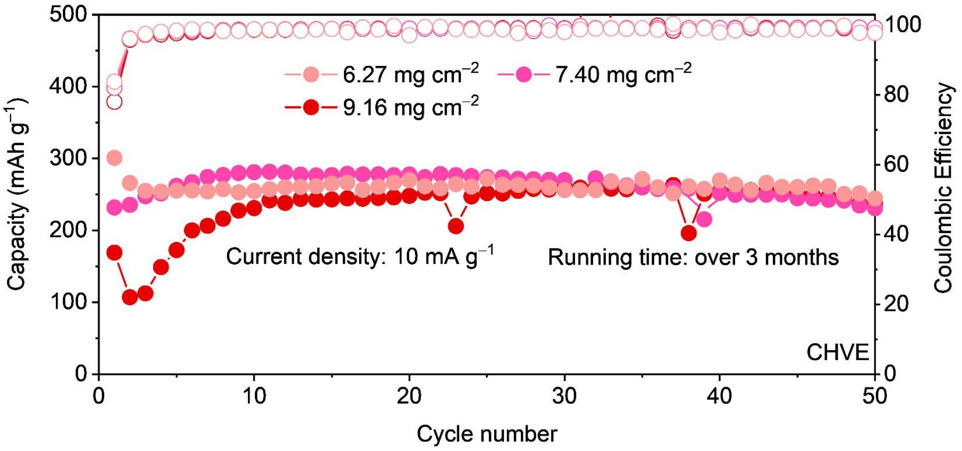

Fig. S27 Cycling performance of high area mass loading graphite electrode in CHVE under a current density of 10 mA g^{-1}.

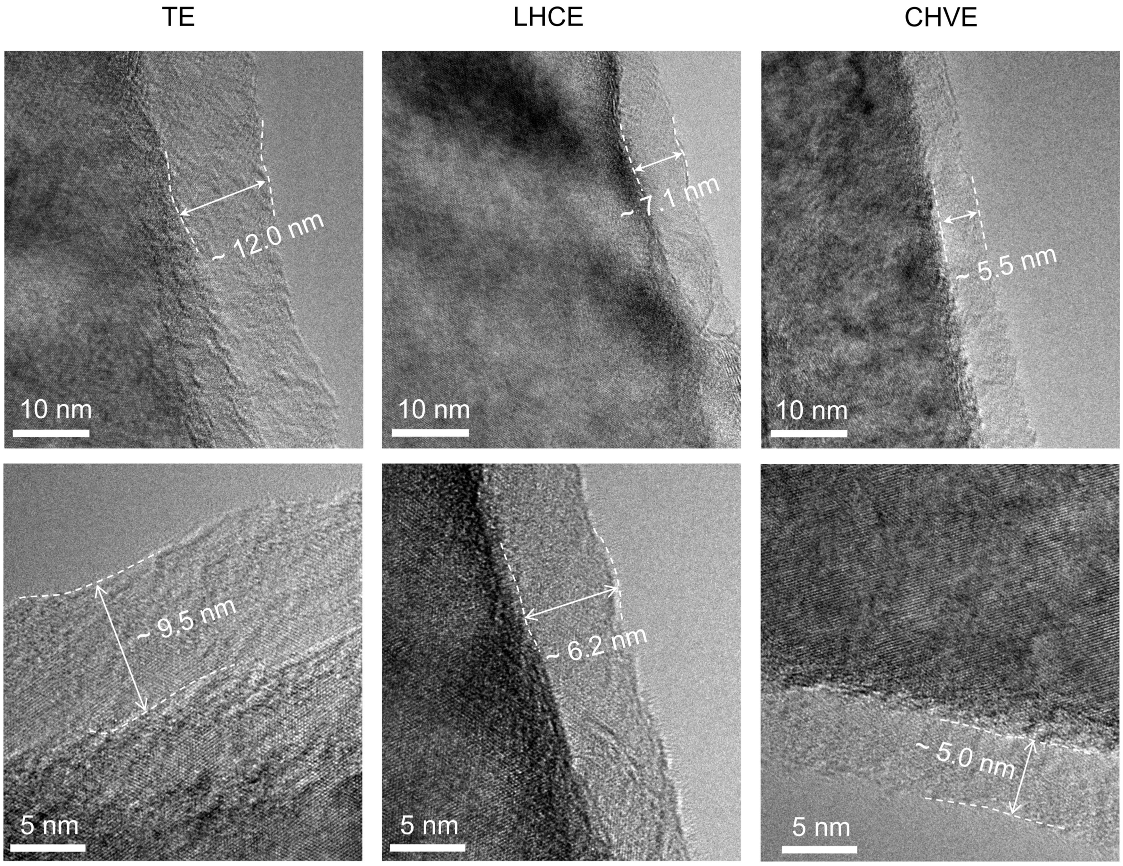

Fig. S28 TEM images of the graphite electrodes after 5 cycles in TE, LHCE and CHVE.

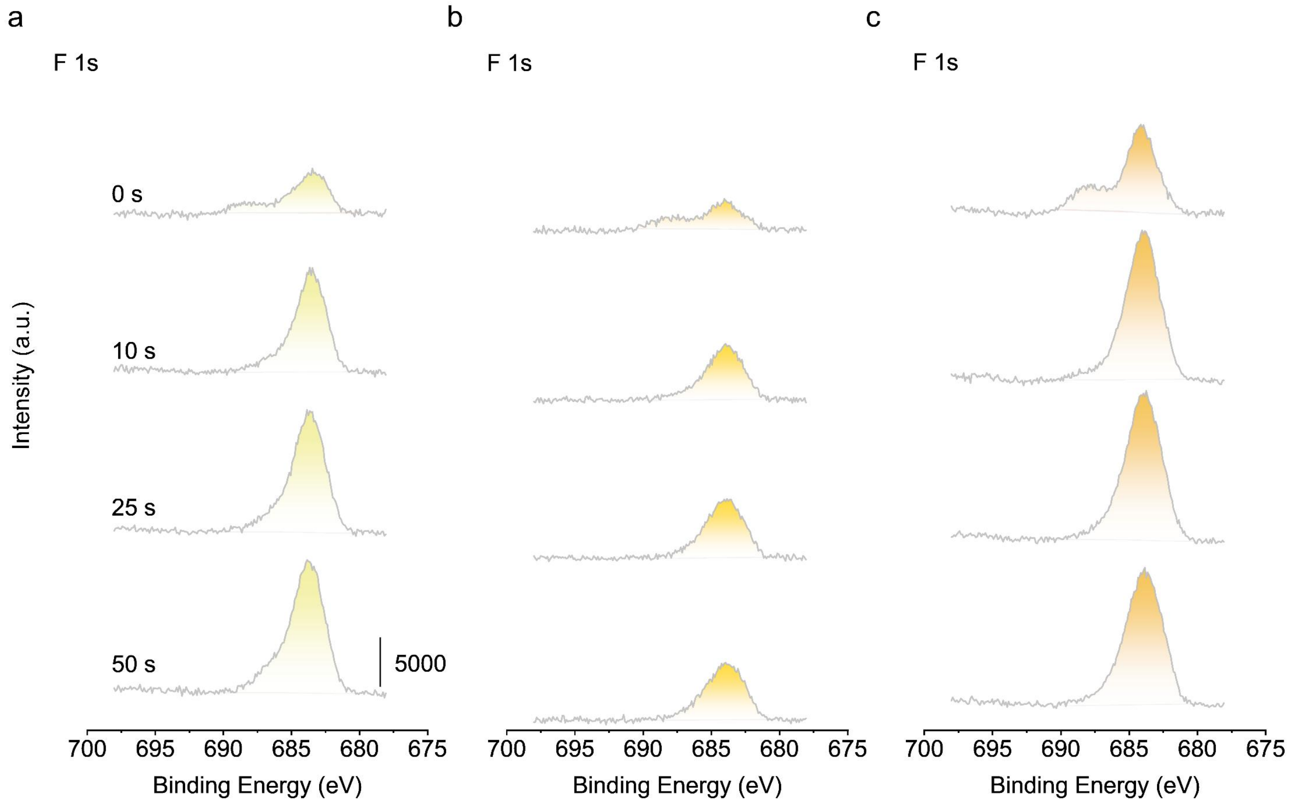

Fig. S29 F1s XPS profiles of graphite electrodes (after 5 cycles) with (a) TE, (b) LHCE and (c) CHVE as a function of sputtering time.

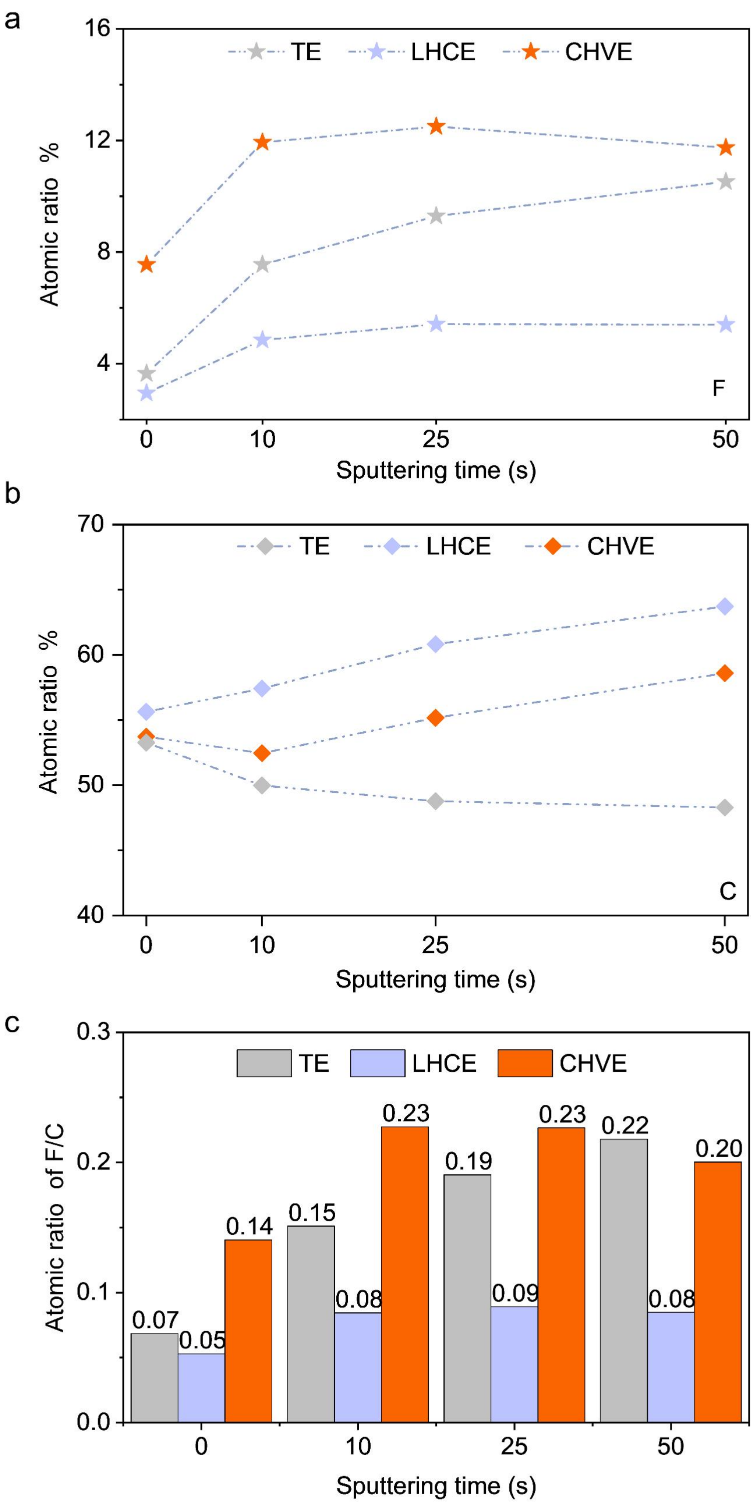

Fig. S30 The atomic ratios of (a) F elementals, (b) C elementals and (c) F: C atomic ratio for graphite electrodes (after 20 cycles) at different sputtering depths.

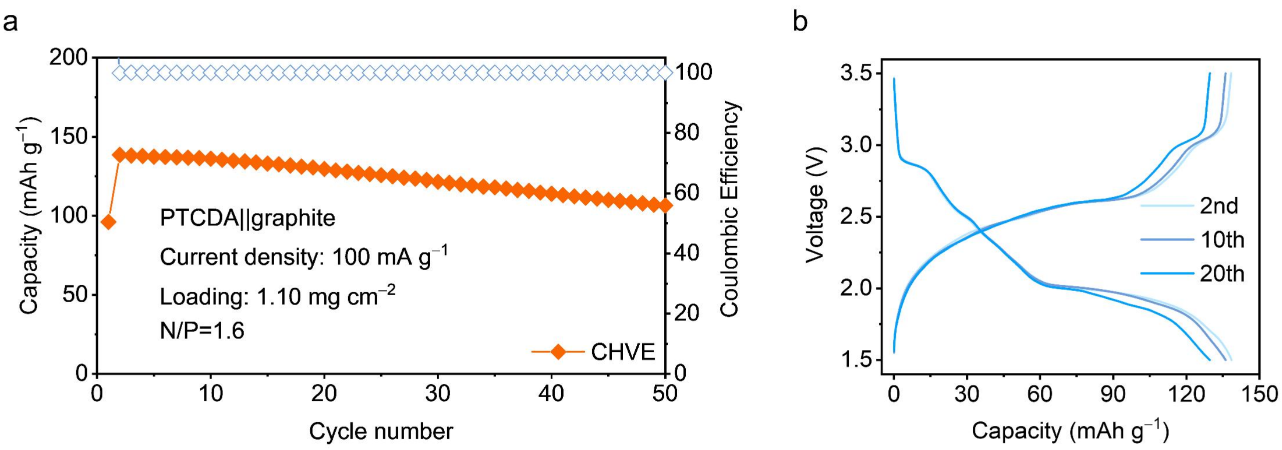

Fig. S31 (a) Cycling performance and (b) charge–discharge curves of PTCDA||graphite full-cell operating in the 1.5–3.5 V range.

Note: Before assembling the full cell, the graphite anode is pre-potassiated. First, the graphite and K foil are assembled into a battery, and cycled thrice. The cell is finally kept in a discharge state at which time potassium ions are intercalated between the graphite layers, completing the pre-potassium process. Subsequently, the graphite electrode is removed from the half-cell and reassembled with the PTCDA cathode into a full-cell. This assembly step ensures that the graphite anode contains a certain amount of K^+ in its initial state, thereby reducing capacity loss during the first charge and discharge process and improving the overall performance and cycling stability of the battery.

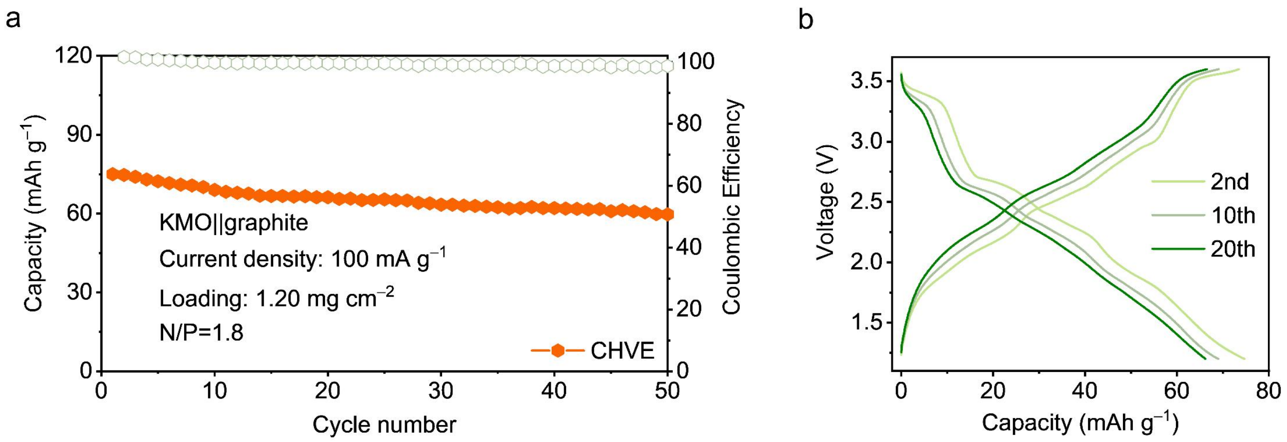

Fig. S32 (a) Cycling performance and (b) charge–discharge curves of KMO||graphite full cell in the 1.2–3.6 V range.

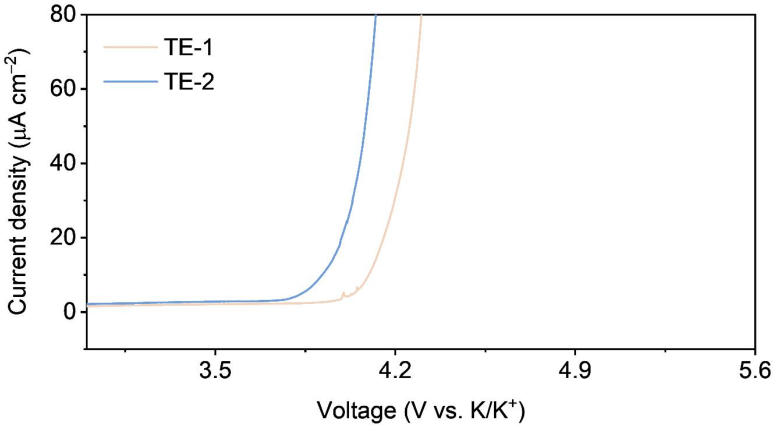

Fig. S33 LSV profiles of 1.5M KFSI in DEE (TE-1) and 1.5M KFSI in DME (TE-2).

Table S1 Three types of electrolyte systems used in this study.

Electrolytes	Salt	Solvent	Diluent/Cosolvent
TE	1.5 M KFSI	DGM (100% v/v)	---
LHCE	1.5 M KFSI	DGM (40% v/v)	TTE (60% v/v)
CHVE	1.5 M KFSI	DGM (16.7% v/v)	DBE (83.3% v/v)

Note: The addition of TTE causes salt precipitation. Therefore, to maintain the consistency of salt concentration, the proportion of TTE was adjusted down to 60%.

Table S2 Average solubility and actual solubility of KFSI in a mixed DGM and TTE solvent in different ratios.

Electrolytes /Solvents	DGM volume ratio	TTE volume ratio	Average solubility (KFSI/M)	Actual solubility (KFSI/M)
LHCE-0.1	0.9	0.1	5.85	5.80
LHCE-0.2	0.8	0.2	5.20	5.00
LHCE-0.3	0.7	0.3	4.55	4.20
LHCE-0.4	0.6	0.4	3.90	3.40
LHCE-0.5	0.5	0.5	3.25	2.60
LHCE-0.6	0.4	0.6	2.60	1.80
LHCE-0.7	0.3	0.7	1.95	1.30
LHCE-0.8	0.2	0.8	1.30	0.80
LHCE-0.9	0.1	0.9	0.65	0.30
TTE	0	1	0	
DGM	1	0	~ 6.50	

Table S3 Average solubility and actual solubility of KFSI in a mixed DGM and DBE solvent in different ratios.

Electrolytes /Solvents	DGM volume ratio	DBE volume ratio	Average solubility (KFSI/M)	Actual solubility (KFSI/M)
CHVE-0.14	0.86	0.14	5.59	5.59
CHVE-0.20	0.80	0.20	5.20	5.38
CHVE-0.26	0.74	0.26	4.82	4.50
CHVE-0.33	0.67	0.33	4.37	4.87
CHVE-0.40	0.60	0.40	3.92	4.34
CHVE-0.50	0.50	0.50	3.28	4.00
CHVE-0.60	0.40	0.60	2.63	3.36
CHVE-0.69	0.31	0.69	2.05	2.92
CHVE-0.75	0.25	0.75	1.66	2.60
CHVE-0.80	0.20	0.80	1.34	2.28
CHVE-0.83	0.17	0.83	1.15	2.17
CHVE-0.93	0.07	0.93	0.50	1.67
DBE	0	1.00	0.05	
DGM	1.00	0	6.50	

Table S4 The average coordination number N(r) of K-O_{DGM}, K-O_{anion}, K-O_{TTE} and K-O_{DBE} in the three electrolytes.

Electrolytes	N(*r*) of K-O_{DGM}	N(*r*) of K-O_{anion}	N(*r*) of K-O_{TTE}	N(*r*) of K-O_{DBE}
TE	7.67	0.82	--	--
LHCE	5.14	2.13	0.01	--
CHVE	2.00	2.35	--	2.84

Table S5 The WAXS results of various KFSI concentrations in the mixed DGM and DBE electrolytes.

Label	DGM Solvent	DBE Cosolvent	KFSI solubility (M)
1G5B	16.7% v/v	83.3% v/v	0
1G5B-0.58			0.58
1G5B-1.15			1.15
1G5B-2.17			2.17
1G4B	20.0% v/v	80.0% v/v	0
1G4B-0.67			0.67
1G4B-1.34			1.34
1G4B-2.28			2.28
1G3B	25.0% v/v	75.0% v/v	0
1G3B-0.83			0.83
1G3B-1.66			1.66
1G3B-2.60			2.60
2G3B	40.0% v/v	60.0% v/v	0
2G3B-1.32			1.32
2G3B-2.63			2.63
2G3B-3.36			3.36
1G1B	50.0% v/v	50.0% v/v	0
1G1B-1.64			1.64
1G1B-3.28			3.28
1G1B-4.00			4.00
3G2B	60.0% v/v	40.0% v/v	0
3G2B-1.96			1.96
3G2B-3.92			3.92
3G2B-4.34			4.34

Table S6 Comparison of K||graphite cycle time and cycle number in different ether-based electrolytes.

Electrolytes	Current Density (mA g^{-1})	Reversible Capacity (mAh g^{-1})	Cycle Time (months)	Cycle Number (N)	Ref.
4.12mol KFSI/DME-TTE	100	~180	~1.0	200	[25]
2M KFSI/DMM	100	~200	~1.3	200	[26]
1M KFSI/DME-CPME	55.8	~220	~1.4	120	[27]
2M KFSI/DEM-DME	100	~200	~6.1	1000	[28]
2.76 m KFSI/DME-HFE	25	200	~6.7	300	[29]
0.91M KFSI/DEECl	50	~270	~10.5	700	[30]
This work	50	230	17	1200	--

Table S7 The broader electrolyte systems composition.

Electrolytes	Salt	Solvent	Diluent/Cosolvent
CHVE-1	1.5 M KFSI	DEE (16.7% v/v)	DBE (83.3% v/v)
TE-1	1.5 M KFSI	DEE (100% v/v)	--
CHVE-2	1.5 M KFSI	DME (16.7% v/v)	DBE (83.3% v/v)
TE-2	1.5 M KFSI	DME (100% v/v)	--
Saturated DEE electrolyte	4.0 MKFSI	--	--
Saturated DME electrolyte	6.8 M KFSI	--	--

REFERENCES

1. Zhang C, Xu Y, Zhou M *et al.* Potassium Prussian blue nanoparticles: a low‐cost cathode material for potassium‐ion batteries. *Adv Funct Mater* 2017; **27**: 1604307.

2. Zhu Y-H, Zhang Q, Yang X *et al.* Reconstructed orthorhombic V2O5 polyhedra for fast ion diffusion in K-ion batteries. *Chem* 2019; **5**: 168-79.

3. Caixiang Z, Hao J, Zhou J *et al.* Interlayer‐engineering and surface‐substituting manganese‐based self‐evolution for high‐performance potassium cathode. *Adv Energy Mater* 2022; **13**: 2203126.

4. Fan L, Ma R, Wang J *et al.* An ultrafast and highly stable potassium–organic battery. *Adv Mater* 2018; **30**: 1805486.

5. Thompson AP, Aktulga HM, Berger R *et al.* LAMMPS - a flexible simulation tool for particle-based materials modeling at the atomic, meso, and continuum scales. *Comput Phys Commun* 2022; **271**: 108171.

6. Kaminski GA, Friesner RA. Evaluation and reparametrization of the OPLS-AA force field for proteins via comparison with accurate quantum chemical calculations on peptides†. *J Phys Chem B* 2001; **105**: 6474-87.

7. Lopes JNC, Shimizu K, Pa´dua AAH *et al.* Potential energy landscape of bis(fluorosulfonyl)amide. *J Phys Chem B* 2008; **112**: 9449–55.

8. Dodda LS, Vilseck JZ, Tirado-Rives J *et al.* 1.14*CM1A-LBCC: Localized bond-charge corrected CM1A charges for condensed-phase simulations. *J Phys Chem B* 2017; **121**: 3864-70.

9. Dodda LS, Cabeza de Vaca I, Tirado-Rives J *et al.* LigParGen web server: an automatic OPLS-AA parameter generator for organic ligands. *Nucleic Acids Res* 2017; **45**: W331-6.

10. Jorgensen WL, Tirado-Rives J. Potential energy functions for atomic-level simulations ofwater and organic and biomolecular systems. *Proc Natl Acad Sci USA*. 2004; **102**: 6665–70.

11. Frisch MJ, Trucks GW, Schlegel HB *et al.* Gaussian 16 Rev. C.01. Wallingford, CT; 2016.

12. Johnson ER, Becke AD. A post-Hartree–Fock model of intermolecular interactions. *J Chem Phys* 2005; **123**: 024101.

13. Grimme S, Antony J, Ehrlich S *et al.* A consistent and accurateab initioparametrization of density functional dispersion correction (DFT-D) for the 94 elements H-Pu. *J Chem Phys* 2010; **132**: 154104.

14. Papajak E, Zheng J, Xu X *et al.* Perspectives on Basis Sets Beautiful: Seasonal Plantings of Diffuse Basis Functions. *J Chem Theory Comput* 2011; **7**: 3027-34.

15. Zheng J, Xu X, Truhlar DG. Minimally augmented Karlsruhe basis sets. *Theor Chem Acc* 2010; **128**:

295-305.

16. Schauperl M, Nerenberg PS, Jang H *et al.* Non-bonded force field model with advanced restrained electrostatic potential charges (RESP2). *Commun Chem* 2020; **3**: 44.

17. Zhang J, Lu T. Efficient evaluation of electrostatic potential with computerized optimized code. *Phys Chem Chem Phys* 2021; **23**: 20323-8.

18. Lu T, Chen F. Multiwfn: A multifunctional wavefunction analyzer. *J Comput Chem* 2011; **33**: 580-92.

19. Leontyev IV, Stuchebrukhov AA. Electronic polarizability and the effective pair potentials of water. *J Chem Theory Comput* 2010; **6**: 3153-61.

20. Park C, Kanduč M, Chudoba R *et al.* Molecular simulations of electrolyte structure and dynamics in lithium–sulfur battery solvents. *J Power Sources* 2018; **373**: 70-8.

21. Martínez L, Andrade R, Birgin EG *et al.* PACKMOL: A package for building initial configurations for molecular dynamics simulations. *J Comput Chem* 2009; **30**: 2157-64.

22. Jewett AI, Stelter D, Lambert J *et al.* Moltemplate: A tool for coarse-grained modeling of complex biological matterand soft condensed matter physics. *J Mol Biol* 2021; **433**: 166841.

23. Qiao Z, Wang X, Zhai Y *et al.* In situ real-time observation of formation and self-assembly of perovskite nanocrystals at high temperature. *Nano Lett* 2023; **23**: 10788-95.

24. Qian K, Winans RE, Li T. Insights into the nanostructure, solvation, and dynamics of liquid electrolytes through small-angle X-ray scattering. *Adv Energy Mater* 2020; **11**: 2002821.

25. Nie P, Liu M, Qu W *et al.* Unravelling the solvation structure and interfacial mechanism of fluorinated localized high concentration electrolytes in K-ion batteries. *Adv Funct Mater* 2023; **33**: 2302235.

26. Ma X, Fu H, Shen J *et al.* Green ether electrolytes for sustainable high-voltage potassium ion batteries. *Angew Chem Int Ed* 2023; **62**: e202312973.

27. Xie H, Liang H, Kumar P *et al.* Intermolecular interaction mediated potassium ion intercalation chemistry in ether-based electrolyte for potassium-ion batteries. *Adv Funct Mater* 2024; **34**: 2401118.

28. Chen W, Zhang D, Fu H *et al.* Restructuring electrolyte solvation by a partially and weakly solvating cosolvent toward high-performance potassium-ion batteries. *ACS Nano* 2024; **18**: 12512-23.

29. Qin L, Xiao N, Zheng J *et al.* Localized high-concentration electrolytes boost potassium storage in high -loading graphite. *Adv Energy Mater* 2019; **9**: 1902618.

30. Hu Y, Fu H, Geng Y *et al.* Chloro-functionalized ether-based electrolyte for high-voltage and stable potassium-ion batteries. *Angew Chem Int Ed* 2024; **63**: e202403269
